# An Anti-MRSA Phage From Raw Fish Rinse: Stability Evaluation and Production Optimization

**DOI:** 10.3389/fcimb.2022.904531

**Published:** 2022-05-17

**Authors:** Israa M. Abd-Allah, Ghadir S. El-Housseiny, Mohammad Y. Alshahrani, Samar S. El-Masry, Khaled M. Aboshanab, Nadia A. Hassouna

**Affiliations:** ^1^Department of Microbiology and Immunology, Faculty of Pharmacy, Ain Shams University, Cairo, Egypt; ^2^Department of Clinical Laboratory Sciences, College of Applied Medical Sciences, King Khalid University, Abha, Saudi Arabia; ^3^Department of Agricultural Microbiology, Faculty of Agriculture, Ain Shams University, Cairo, Egypt

**Keywords:** bacteriophage, MRSA, resistance, stability, response surface methodology

## Abstract

Accumulating evidence has denoted the danger of resistance in tenacious organisms like methicillin-resistant *Staphylococcus aureus* (MRSA). MRSA, a supple bacterium that adopts a variety of antibiotic resistance mechanisms, is the cause of multiple life-threatening conditions. Approaching a post-antibiotic era, bacteria-specific natural predators, bacteriophages, are now given the chance to prove eligible for joining the antibacterial weaponry. Considering the foregoing, this study aimed at isolating bacteriophages with promising anti-MRSA lytic activity, followed by characterization and optimization of the production of the bacteriophage with the broadest host range. Five phages were isolated from different environmental sources including the rinse of raw chicken egg, raw milk, and, remarkably, the raw meat rinses of chicken and fish. Examined for lytic activity against a set of 23 MRSA isolates collected from various clinical specimens, all five phages showed relatively broad host ranges with the bacteriophage originally isolated from raw fish rinse showing lytic activity against all the isolates tested. This phage is suggested to be a member of Siphoviridae family, order Caudovirales, as revealed by electron microscopy. It also exhibited good thermal stability and viability at different pH grades. Moreover, it showed reasonable stability against UV light and all viricidal organic solvents tested. Optimization using D-optimal design by response surface methodology was carried out to enhance the phage yield. The optimum conditions suggested by the generated model were a pH value of 7, a carbon source of 0.5% w/v sucrose, and a nitrogen source of 0.1% w/v peptone, at a temperature of 28°C and a bacterial inoculum size of 10^7^ CFU/ml, resulting in a 2 log-fold increase in the produced bacteriophage titer. Overall, the above findings indicate the lytic ability inflicted by this virus on MRSA. Apparently, its stability under some of the extreme conditions tested implies its potential to be a candidate for pharmaceutical formulation as an anti-MRSA therapeutic tool. We hope that bacteriophages could tip the balance in favor of the human front in their battle against multidrug-resistant pathogens.

## 1 Introduction

The Universe is full with creatures; they are all related one way or another. Looking at their sizes, the tiniest organisms have an incommensurable impact onto human beings. They present an impressive dichotomy in their influence; some are foes, while others could be friends. No matter how else one may look at modern medicine, the past century has witnessed the subjugation of noxious bacteria, the cause of infectious diseases, and this has altered the fate of the global populace. Antibiotics, the miraculous drugs, have rescued entire communities from the danger of bacterial outbreaks, they saved the lives of immunocompromised individuals and neonates, and they enabled physicians to surgically operate on patients, unhesitatingly. Not only these, antibiotics also empowered the application of cancer therapies. In addition, the good of antibiotics did not stop at the human realm; conversely, it extended to serve non-medical purposes like increasing the efficiency of livestock, food preservation, aquaculture, and some other industries (Meek et al., 2015; Dodds, 2017; Waglechner and Wright, 2017; Wencewicz, 2019; Gao and Zhang, 2021).

Unluckily, antibiotics have been steadily stripped off their power, and the antimicrobial resistance (AMR) havoc has been globally encountered for a long time now (Gao and Zhang, 2021; Montassier et al., 2021). The future is further gloomed by the progressive expansion of resistance even across familiar pathogens like *Staphylococcus aureus*. Soon after methicillin was implemented as the first beta-lactamase-resistant agent in 1959, cultures of methicillin-resistant *Staphylococcus aureus* (MRSA) were detected in hospitals and, few decades later, in community and livestock as well (Grema, 2015).

MRSA is known to cause multifarious, hard-to-treat diseases like diabetic foot and surgical-site infections, pneumonia, sepsis, joint, and multiple skin infections (Grema, 2015; Craft et al., 2019; MRSA | CDC, 2019; Algammal et al., 2020; Siddiqui and Koirala, 2020). Besides, intestinal colonization with MRSA has contributed to the risk of horizontal gene transfer to commensal organisms and also led to increasing MRSA-associated colitis (Nataraj and Mallappa, 2021). As declared by the Infectious Diseases Society of America (IDSA), *S. aureus* is a member of the organisms grouped as “ESKAPE” connoting its ability to evade even the last-resort antibiotics such as vancomycin (Santajit and Indrawattana, 2016). Furthermore, in 2017, The World Health Organization (WHO) designated MRSA as a high priority multidrug-resistant (MDR) pathogen (WHO, 2017).

“If we fail to act, we are looking at an almost unthinkable scenario where antibiotics no longer work and we are cast back into the dark ages of medicine”—by this statement, David Cameron, the former UK Prime Minister, has emphasized the need for a coordinated and immediate counterattack against antimicrobial resistance (Jim O’Neill, 2016; Raina, 2019). Urgent execution of a coherent action plan is a priority not to evoke such a dreadful future where the aspiration of sometimes the entire population is curtailed to survival in the face of a set of otherwise easily treatable diseases (Meek et al., 2015; Dodds, 2017).

One way apt to supplement the suggested combatting policies is calling out for a centenarian that has the potential to be an added value to the antibacterial weaponry, bacteriophage. Bacteriophage is the bacteria-specific virus that is capable of destroying the bacterial cells it breeds on, especially the lytic ones (Ackermann, 2012). In 1926, in a book called *The Bacteriophage and its Behaviour*, D’Herelle described the successful use of bacteriophages for treatment of various infections (D’Herelle, 1929). After that, some companies like Abbott and Eli Lilly implemented the use of therapeutic phages against staphylococcal infections and proved quite promising (Ackermann, 2012).

Hitherto, the use of bacteriophages for the treatment of resistant bacterial infections has regained some interest over the past few decades, and some advancements have been accomplished as well in this regard (Luong et al., 2020). On that account, this study sought the isolation of bacteriophages active against clinical isolates of MRSA, as an example of a dangerous and common multidrug-resistant organism, in a trial to highlight the potential that phages possess in the battle against bacterial resistance (Berryhill et al., 2021; Erol et al., 2021; Gordillo Altamirano and Barr, 2021). It also aimed at evaluating the *in vitro* efficacy and stability of the promising phage. Moreover, it attempted maximizing the production of this phage, applying different modalities.

## 2 Materials and Methods

### 2.1 Bacterial Isolates: Collection, Identification, and Susceptibility Testing

Twenty-three MRSA isolates were collected from the grown cultures of several clinical specimens in the Diagnostic Microbiology Laboratories in two University Hospitals, Cairo, Egypt. To confirm their identification, the isolates grown on mannitol salt agar (Kateete et al., 2010) were subjected for microscopical examination and biochemical tests, namely, coagulase and catalase (Fisk, 1940; Clarke and Cowan, 1952; Brown et al., 2005; Gayar et al., 2014). In addition, the isolates were introduced in the VITEK2 automated system (Shetty et al., 1998). They were also tested for their susceptibility to cefoxitin using disk diffusion method (Patel and Clinical and Laboratory Standards Institute, 2017; Craft et al., 2019). The 23 MRSA isolates were deposited in the culture collection of Ain Shams University of the World Data Centre for Microorganisms (WDCM) (http://ccinfo.wdcm.org/collection/by_id/1186) under the accession codes from CCASU-MRSA-2022-1 to CCASU-MRSA-2022-23. They were preserved as stock aliquots with glycerol at −80°C and were regularly renewed by subculture on nutrient agar slants as well (Hubálek, 2003; Prakash et al., 2013). Their susceptibility profiles towards some anti-MRSA agents (amikacin, vancomycin, linezolid, SMX/TMP, doxycycline, and tigecycline) were determined as well using Kirby–Bauer disk diffusion method (Ferraro, 2000; Felten et al., 2002; Shibabaw et al., 2014). The interpretation of the results was done referring to the standard breakpoints of Clinical and Laboratory Standards Institute (CLSI) guidelines 2018 (Weinstein, 2018; CLSI in 2018).

### 2.2 Recovery of Bacteriophages From Environmental Samples

#### 2.2.1 Isolation of *S. aureus*-Specific Bacteriophages

Eighty-eight samples were collected from various environmental sources and deidentified human urine and stool samples collected from the Diagnostic Microbiology Laboratories of Specialized Ain Shams University Hospitals, Cairo, Egypt. This study was approved by Faculty of Pharmacy Ain Shams University Ethics Committee Number, ACUC-FP-ASU RHDIRB2020110301 REC #68. The sources of the respective specimens were chosen based on the possibility of incorporating *S. aureus* and hence bacteriophages specific against it (**Table 1**) (Clokie and Kropinski, 2009; Wommack et al., 2009; Hallajzadeh et al., 2019; Hyman, 2019; Moodley et al., 2019; Pertics et al., 2020). All samples were stored at 4°C until time of processing. Liquid samples were handled according to their visible clarity. Visibly clear samples were used as such. Turbid samples were exhaustively filtered using wetted cotton and filter paper. Only the clear supernatant was kept for further processing (Clokie and Kropinski, 2009; Gill and Hyman, 2010; Hallajzadeh et al., 2019; Mahmoud et al., 2020). One MRSA isolate was used as the bacterial host for isolation of bacteriophages. A loopful from the bacterial isolate grown onto nutrient agar slant was inoculated into tryptic soy broth (TSB) and incubated for 6–7 h in a shaking water bath at 37°;C, 180 rpm. The suspension was used when heavily turbid, with optical density equivalent to a bacterial count of 10^9^ CFU/ml (Gayar et al., 2014). For phage isolation, TSB was prepared as double strength broth, especially supplemented with traces of calcium carbonate and magnesium sulfate heptahydrate (Serwer et al., 2004; El-Dougdoug et al., 2019; Ács et al., 2020). A mixture of the bacterial host inoculum, the environmental sample, and the isolation medium at a ratio of 1:1:10, respectively, was incubated overnight at 28°;C, 180 rpm (Hussein et al., 2019). Next day, the co-culture was centrifuged for 20 min at 6,000 rpm. Chloroform was added to the supernatant at 1:10 ratio and was vigorously shaken for 5 min (Stenholm et al., 2008; Hussein et al., 2019). The suspensions were left to separate for 4–6 h at 4°;C, and the supernatant formed above a plug-like sediment was collected and recentrifuged under the same conditions. The harvested lysates were stored at 4°C.

**Table 1:** A list of the environmental samples collected for the isolation of bacteriophages and the results of the spot test screening of the lysates obtained.

Source	Number	Initially positive spot test	Consistently positive spot test
Ponds and lakes	5	1	-
Sea water	2	2	-
River water and landfill leachate	2	-	-
Domestic tap water	3	1	-
Hospital and laboratories’ drainages	3	3	-
Rinse of leafy vegetables	3	-	-
Poultry feathers, litter and raw chicken rinses	12	8	2: L10, L12
Sewage samples	12	5	-
Soil samples	5	-	-
Raw milk samples	10	4	1: H10
Wastewater draining from different food stores and restaurants	10	2	-
Rinses of raw chicken eggs	3	1	1: D3
Raw meat and fish rinses	5	2	1: F2
Human urine and stool samples	13	-	
Total	88	29	5

#### 2.2.2 Screening of the Obtained Lysates for Lytic Activity Against MRSA

The fresh lysates were qualitatively screened for anti-MRSA bacteriophages *via* spot test. It was performed according to the technique originally described by Adams (1959). Clear inhibition spots indicated the presence of phages active against MRSA (Adams, 1959; Sambrook et al., 1989; Clokie and Kropinski, 2009). The lysates with consistently positive spot test results were proceeded for the quantitative plaque assay. It was performed using the standard double agar overlay (DAO) technique (Anderson et al., 2011; Vahedi et al., 2018). On the following day, plaques were examined and counted. The phage titer was calculated using the following equation (Clokie and Kropinski, 2009):


Phage titer in plaque−forming unit per ml (PFU/ml)=number of plaques/(volume of lysate infected×dilution)

#### 2.2.3.Phage Propagation

The same procedure used for isolation was repeated thrice, but the starting sample was replaced by an aliquot from the obtained crude lysate. In essence, propagation was regularly performed in order to have a sizeable stock of high-titer phage suspensions.

### 2.3 Evaluation of Some Characteristics of the Isolated Bacteriophage F2

#### 2.3.1 Host Range

The lysates (selected based on their consistent spot test positive result) were examined for lytic activity against the remaining 22 MRSA isolates as bacterial indicators using spot test (Mahmoud et al., 2020). The lysate showing the best host range was selected for further studies.

#### 2.3.2 Morphology of the Isolated *S. aureus* Phage Particles

Preparing for microscopical examination, a concentrated phage suspension of the selected lysate was centrifuged at 10,000 rpm for 25 min twice in a row and was then filtered using a syringe filter (0.22 μm). After that, a 20-μl sample was prepared as directed by Kalatzis et al. and was examined using a transmission electron microscope (JEOL_JEM_1010 Electron Microscope Siemens & Halske, Germany, performed at Regional Center for Mycology and Biotechnology, Al-Azhar University, Cairo, Egypt) (Ackermann, 1998; Kalatzis et al., 2016; Nasser et al., 2019; Mahmoud et al., 2020).

#### 2.3.3 Longevity Test

An aliquot of the phage lysate was kept at temperatures of 4°C, 37°C, and −20°C for 90 days. Each was examined for infectivity at days 1, 2, 3, 4, 5, 6, 7, 15, 30, 60, and 90 by spot test (Fortier and Moineau, 2009; Mahmoud et al., 2020).

#### 2.3.4 Thermal Stability

A fixed volume of the phage suspension was exposed to a temperature range from 30°C to 60°;C at 5°; intervals in a previously adjusted water bath for 1 h. An aliquot was aspirated at the end of the exposure time and was immediately examined for loss of infectivity by spot test. Plates were examined after incubation, and thermal inactivation point was determined accordingly (Mahmoud et al., 2020).

#### 2.3.5 pH Stability

One milliliter of the phage suspension was added to 1 ml TSB previously adjusted to a specific pH in the range from 1 to 13 using 1N NaOH and HCl. Mixtures were left at room temperature for 1 h. The infectivity of the phage at the different pH values was assessed using the standard spot test (Jamalludeen et al., 2007; Mahmoud et al., 2020).

#### 2.3.6 Stability Towards UV Light

A sample of the phage suspension was directly irradiated by the UV light into a laminar flow hood at a constant 30-cm distance from the source for 1 h total exposure time. Samples were drawn at different intervals of 10, 20, 30, 40, 50, and 60 min and were spotted for infectivity (Rodriguez et al., 2014; Alič et al., 2017; Kim et al., 2018).

#### 2.3.7 Sensitivity to Organic Solvents

Three solvents, namely, chloroform, ethanol, and isopropyl alcohol, were diluted with distilled water to final concentrations of 10, 30, and 50% v/v; 100% v/v concentration of each was used as well. An aliquot of the phage suspension was blended with each of these concentrations in 1:1 ratio and was incubated for 1 h at room temperature (Kim et al., 2018; Łubowska et al., 2019). The effect was assessed using spot test.

### 2.4 Optimization of the Anti-MRSA Phage Production

#### 2.4.1 Studying Different Factors Influencing the Production of the Phage F2 One Factor at a Time

The effect of different factors on the co-culture functionality was tested individually, and the response in terms of the produced phage titer (PFU/ml) was assessed at the end of each run carrying out the standard plaque assay. The factors selected were production medium components such as carbon source (0.5% v/v glycerol and 0.5% w/v sucrose) and nitrogen source (0.1% w/v peptone and 0.1% w/v glycine) (Kim et al., 2021), the bacterial host inoculum size (10^7^, 10^8^, and 10^9^ CFU/ml), and temperature (28°C, 33°C, and 37°C) (Grieco et al., 2012; González-Menéndez et al., 2018a; Reuter and Kruger, 2020). Optimal factors that led to the highest phage production were selected for further experiments.

#### 2.4.2 Production Optimization Using Response Surface Methodology

The factors selected to be optimized using response surface methodology (RSM) were pH (coded A), sucrose concentration (coded B), and peptone concentration (coded C). Each factor was incorporated at three different levels (**Table 2**). The design of experiments (DOE) was performed using D-optimal design, with the help of the statistical software package, Design Expert^®^ v. 7.0 (Design Expert Software, Stat-Ease Inc., Statistics Made Easy, Minneapolis, MN, USA). Seventeen production runs were carried out as proposed by the software (**Table 3**). One response value was monitored at the end of each run: phage titer in PFU/ml. The relationship between the factors was determined, and a polynomial cubic equation was fitted by the software using the data obtained from the experiments (Bhaumik et al., 2013; González-Menéndez et al., 2018a; El-Housseiny et al., 2021).

**Table 2:** The factors selected for RSM optimization of phage production and their levels.

	Factor	Level		
		-1	0	+1
Media components	A: pH	6	7	8
	B: Carbon source; sucrose concentration (%w/v)	0.1	0.5	0.9
	C: Nitrogen source; peptone concentration (%w/v)	0.05	0.1	0.15

A, pH; B, carbon source; C, nitrogen source.

**Table 3:** The D-optimal design production runs suggested for phage F2 production and their observed responses.

Run	A: pH	B: Sucrose (% w/v)	C: Peptone (% w/v)	Response observed (phage count) (PFU/ml)
**1**	6	0.10	0.05	3×10^10^
**2**	6	0.9	0.15	6×10^11^
**3**	7	0.10	0.10	3.8×10^11^
**4**	8	0.9	0.15	1.3×10^12^
**5**	7.5	0.7	0.10	6.6×10^11^
**6**	6	0.10	0.15	4.5×10^10^
**7**	6	0.10	0.15	4.2×10^10^
**8**	6	0.10	0.05	2.7×10^10^
**9**	8	0.5	0.05	7.2×10^11^
**10**	8	0.10	0.15	5.4×10^10^
**11**	7	0.90	0.05	6.4×10^11^
**12**	6	0.90	0.05	2×10^10^
**13**	7	0.50	0.10	2.2×10^12^
**14**	8	0.90	0.15	1.4×10^12^
**15**	7	0.50	0.15	9×10^10^
**16**	8	0.10	0.05	8.6×10^9^
**17**	6	0.50	0.10	2.6×10^11^
**Control**	7	–	–	3×10^10^

##### 2.4.2.1 Statistical Analysis

Replicates of each run were carried out, and the calculated mathematical mean was considered the observed result. Data analysis was performed by Design Expert^®^ v. 7.0. Response surfaces and model diagnostic plots were also generated using the same program (El-Sayed et al., 2020a).

##### 2.4.2.2 Verification of the RSM Optimization Results

The numerical optimization function in the Design Expert^®^ Software was used to identify the co-culture conditions optimum for maximum phage production as recommended by the program. An additional experiment was conducted with the newly suggested optimal conditions. The phage titer produced applying these conditions was compared to that obtained under the unoptimized ones.

## 3 Results

### 3.1 Collection, Identification, and Susceptibility Testing of MRSA Isolates

**Figure 1:**
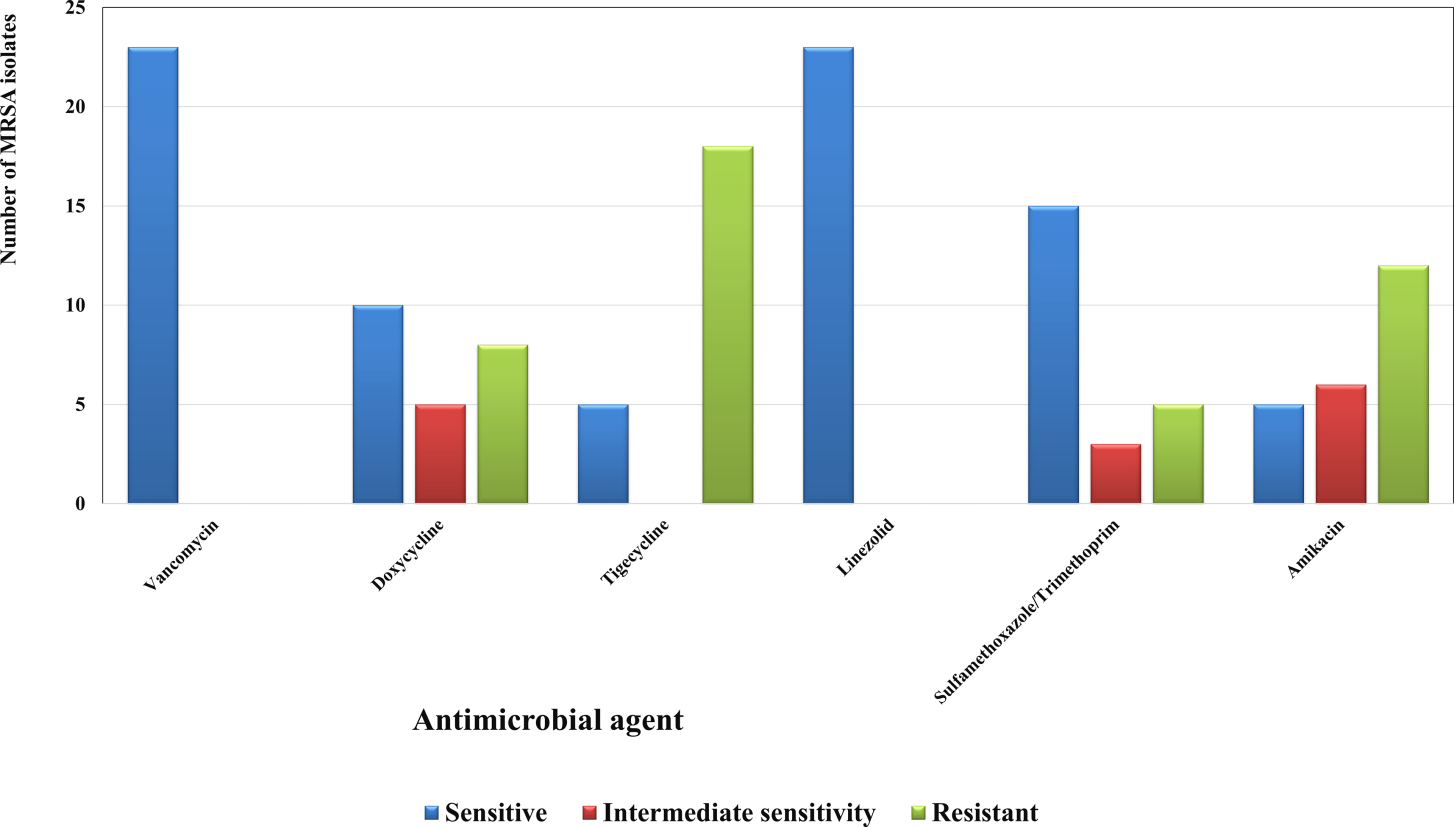
Susceptibility patterns of the collected MRSA isolates (n=23) towards some antimicrobial agents. Each result is the mean of three replicates.

The 23 isolates were identified as *S. aureus* using preliminary identification tests and confirmed by VITEK2. They all showed resistance to cefoxitin, hence proving to be MRSA (Patel and Clinical and Laboratory Standards Institute, 2017; Weinstein, 2018; Craft et al., 2019; Siddiqui and Koirala, 2020). The susceptibility patterns displayed that most of the isolates were resistant to nearly all the tested agents except for vancomycin and linezolid (**Figure 1**).

### 3.2 Recovery of *S. aureus* Bacteriophages and Screening for Activity Against MRSA

**Figure 2:**
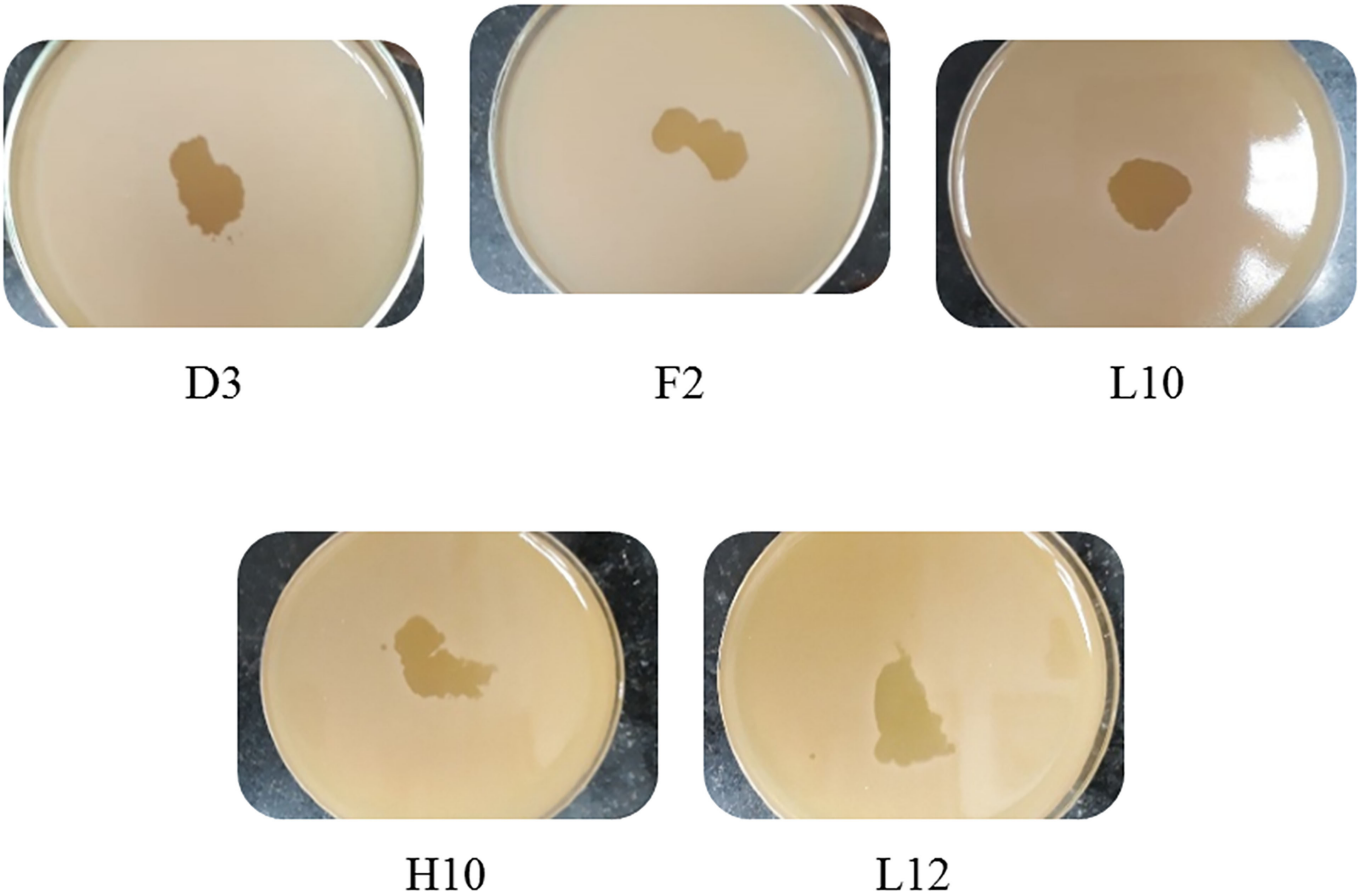
Spot tests of the five harvested lysates against MRSA, showing clear spots and proving their lytic abilities.

**Figure 3:**
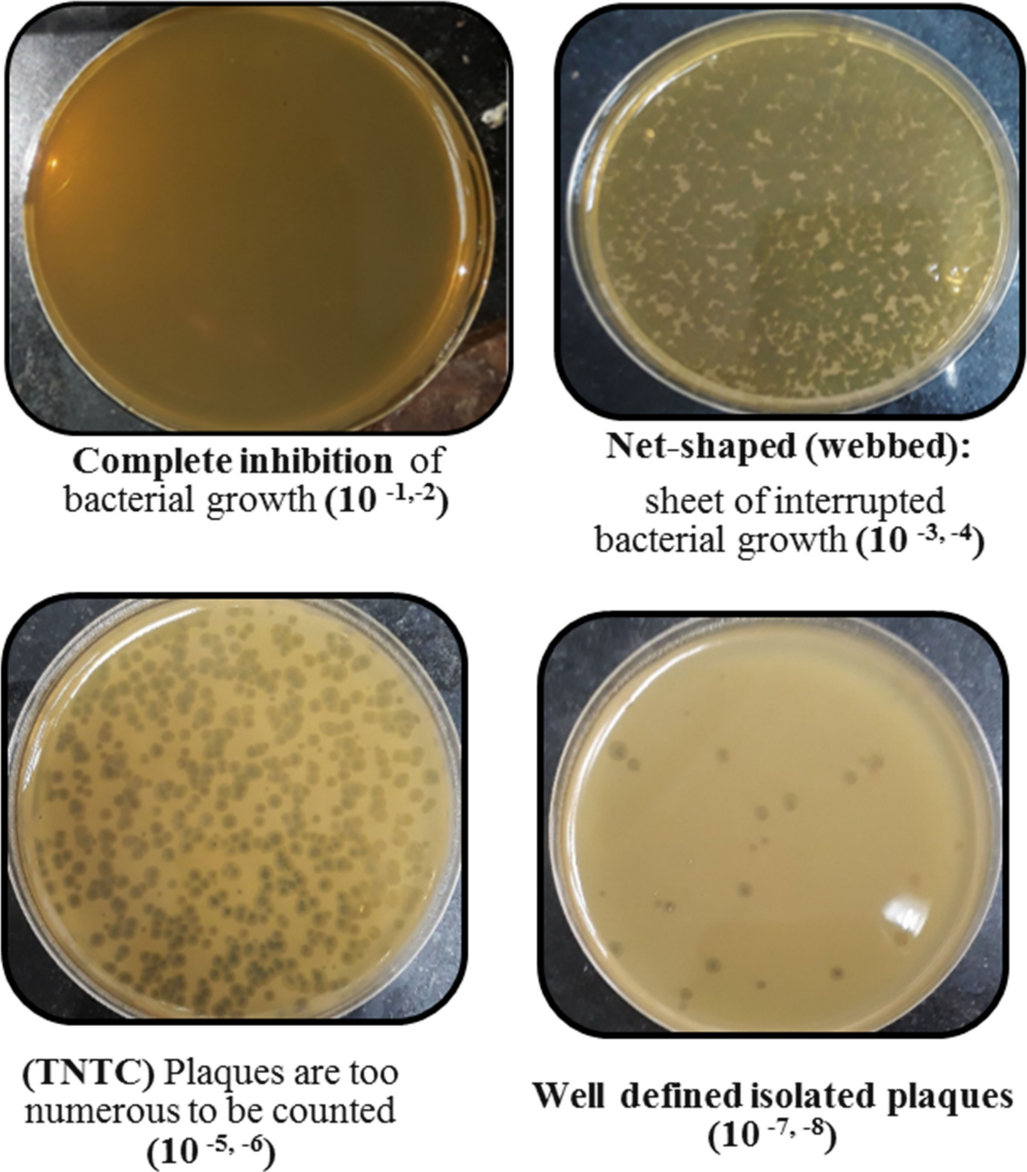
Plaque assay results of phage F2 at different dilutions.

**Figure 4:**
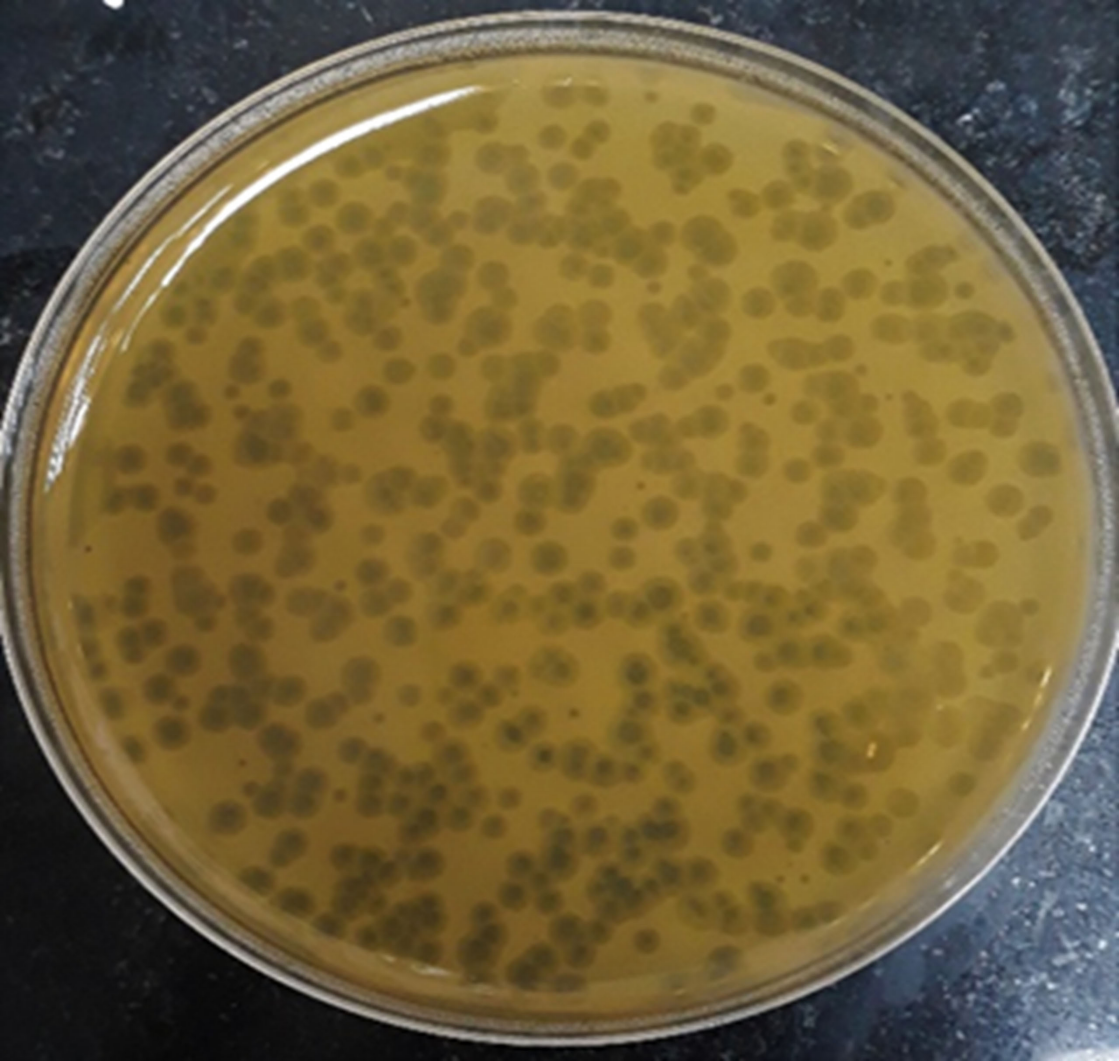
Plaque morphology as presented by phage F2. Plaques are clear, regularly circular, small in size (diameter of 3–5 mm), and halos are shown around the plaques.

Screening resulted in 29 samples showing preliminarily positive spot test against MRSA; however, only five of these lysates showed consistently positive spot tests (**Table 1**; **Figure 2**) and hence were selected for further studies. All lysates had relatively reproducible, high initial titers as determined by plaque assay (>10^8^ PFU/ml) (**Figure 3**). Plaques appeared clear, regularly circular, and small in size (1–5 mm). Interestingly, F2 plaques were surrounded by halos (**Figure 4**).

### 3.3 Evaluation of the Isolated Putative Bacteriophages’ Characters

#### 3.3.1 Host Range

The five lysates were fairly active against most of the isolates. Putative phage F2 showed lytic activity against all the 22 MRSA isolates tested, so it was selected to complete the study.

#### 3.3.2 Morphology of Phage F2 as Demonstrated by TEM

**Figure 5:**
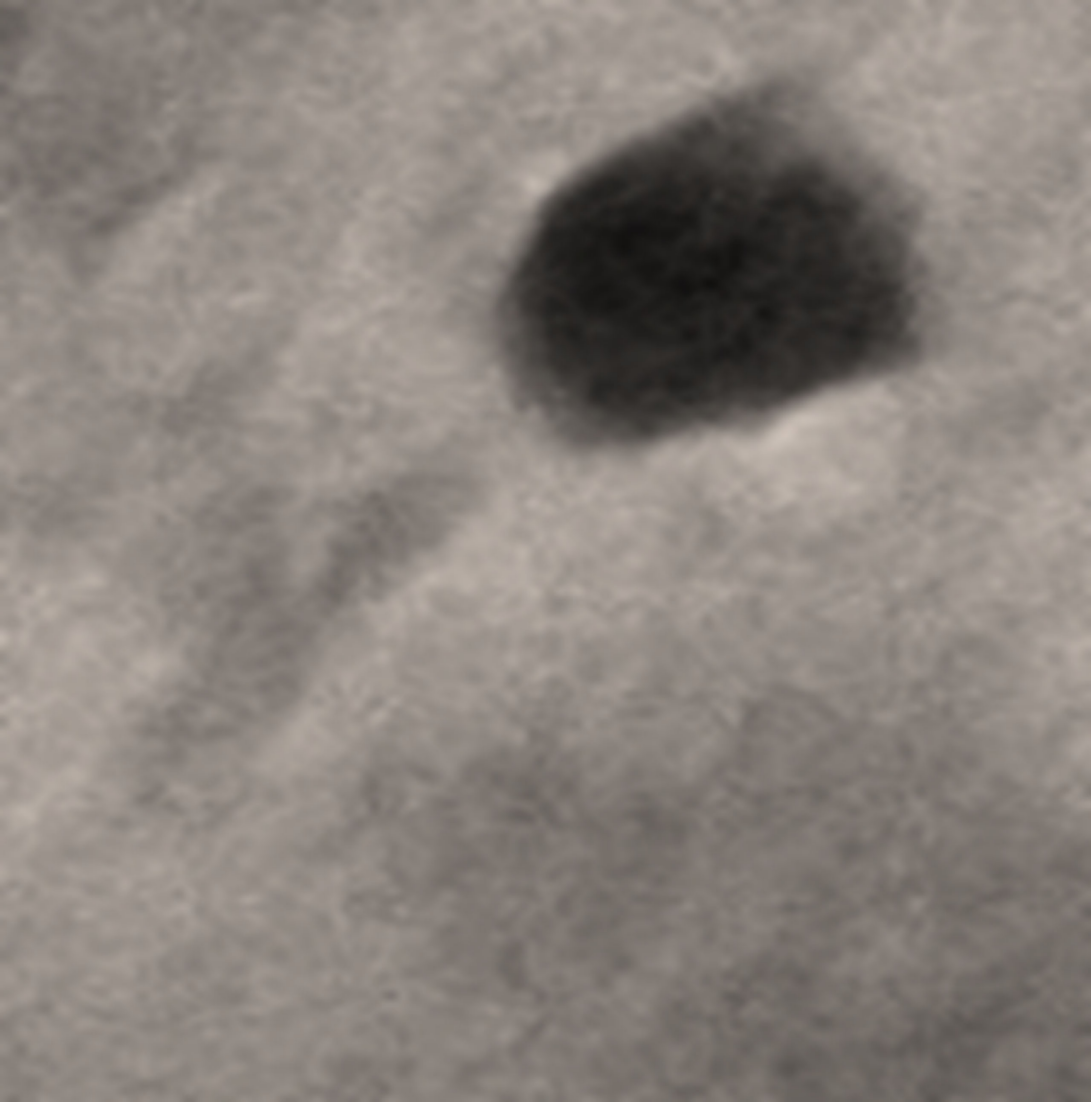
Electron micrograph of bacteriophage F2. The head diameter is 34 nm, and the tail length is 100 nm.

The electron micrograph showed that F2 is a tailed bacteriophage (**Figure 5**), so it is believed to be of the order Caudovirales. The exact tail dimensions were hard to accurately determine because whole tails were scarce in the examined field and the size was highly dependent on the bacteriophage position on the grid. However, the tail size was estimated to be 100 nm, and the head diameter was 34 nm. The tail showed neither head-to-tail connectors nor base plates. Matching these observations to the information found on “Viral Zone” website (Siphoviridae—an overview | ScienceDirect Topics, 2011; Siphoviridae ~ ViralZone, 2011; King et al., 2012) together with the rules compiled by the International Committee on Taxonomy of Viruses (ICTV) (King et al., 2011; King et al., 2012), it could be suggested that F2 might be a member of the Siphoviridae morphotype.

#### 3.3.3 Longevity Test

The F2 aliquots kept at the different temperatures of 4°C, 37°C, and −20°C succeeded to sustain infectivity till the end of the test (90 days).

#### 3.3.4 Thermal Stability

Phage F2 retained its activity along all the temperatures tested except at 60°C, at which the lytic spot completely disappeared. Hence, 60°C was considered its thermal inactivation point.

#### 3.3.5 pH Stability

Along the pH range from 3 to 11, phage F2 was capable of producing clear lytic spots. However, when placed in an environment that is either extremely acidic (pH = 1, 2) or extremely alkaline (pH = 12, 13), the lytic spot disappeared.

#### 3.3.6 Sensitivity to UV

The lytic activity of phage F2 was retained up till 30 min of exposure to UV irradiation. However, it started to gradually decline after this point in time as presented by a weak degradative spot, till it completely disappeared after 1 h of exposure.

#### 3.3.7 Sensitivity to Organic Solvents

The bacteriophage F2 was stable at all the concentrations of the three chemical agents applied, except that its lytic activity weakened with ethanol starting from a concentration of 50% v/v.

### 3.4 Production Optimization of Phage F2

#### 3.4.1 OFAT Optimization

**Figure 6:**
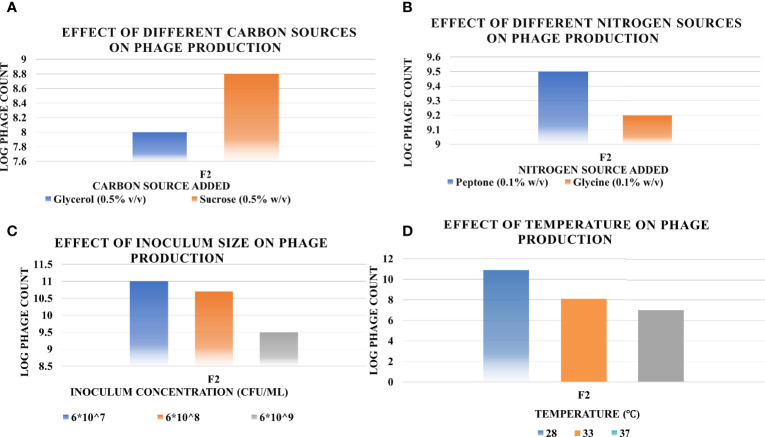
Effect of different factors on the phage production titers using one-factor-at-a-time (OFAT) method, and the optimum level of each that led to maximum phage production (**A**, carbon source; **B**, nitrogen source; **C**, inoculum size; **D**, temperature). Results are the mean of the three replicates.

The results of the OFAT optimization experiments are summarized in **Figure 6** as follows.

a. Effect of Different Carbon Sources
Sucrose at 0.5% w/v resulted in 56-fold increase in titer, while glycerol at the same concentration did not lead to any remarkable increase.
b. Effect of Different Nitrogen Sources
The phage yield increased hugely when applying peptone at 0.1% w/v, approximately 300-fold. However, no noticeable increase occurred when glycine was applied at the same concentration.
c. Effect of Bacterial Inoculum
The host inoculum concentration that led to maximum production of phage F2 was equal to 6×10^7^ CFU/ml.
d. Effect of Temperature
The optimum incubation temperature was 28°;C, resulting in phage titer as high as ~10^10^ PFU/ml.

#### 3.4.2 RSM Optimization

##### 3.4.2.1 The Combined Effect of pH, Carbon, and Nitrogen Sources

The 17 experiments proposed by the Design Expert^®^ Software along with their observed results are shown in **Table 3**. The generated reduced cubic model for the prediction of the produced phage titer (PFU/ml) was as follows:

**Log10 (Phage count)** =-274.83010+83.83276*A+101.56114*B+2065.24586* C-30.75049 * AB-610.77648* AC+16.35854* BC-6.03935* A^2^+13.17374*B^2^+104.52059* C^2^+2.43068* A^2^ B+43.85170* A^2^C-3.12607 * AB^2^

##### 3.4.2.2 Statistical Analysis

To determine the significance of the model and its appropriateness for phage titer prediction, ANOVA was performed (**Table 4**). Both F- and p-values were calculated to represent the significance of the model and its terms. The F-value of the model was 975.10 (p-value = 0.0001), inferring that the model is significant. Regarding the model parameters, the linear terms, namely, A, B, and C, together with the interactive ones, AB, AC, BC, A^2^, B^2^, C^2^, A^2^ B, A^2^ C, and AB^2^, all proved significant, as they had p-values <0.05 (El-Sayed et al., 2020a; El-Sayed et al., 2020b). The coefficient of variation (C.V.%) was 0.25. A C.V.% this low bespeaks the reliability of the experimental data (El-Housseiny et al., 2016; El-Sayed et al., 2020a; El-Sayed et al., 2020b; El-Housseiny et al., 2021). How well the model describes the data was assessed by R^2^, the coefficient of determination. It was 0.9997, indicating that the model can explain 99.97% of the variability that may result in response (El-Sayed et al., 2020a; El-Housseiny et al., 2021). Predicted R-squared (Pred R^2^) was found to be 0.9504 and was tenably in agreement with its adjusted analogue (adj R^2^) of 0.9986. Adequate precision ratio was 97.680, way greater than 4, indicating that this model can be used to navigate the design space (El-Sayed et al., 2020a; El-Housseiny et al., 2021).

**Table 4:** ANOVA for response surface reduced cubic model (F2).

Source	Sum of Squares	df*	Mean Square	F-value	p-value	
Model	9.16	12	0.76	975.10	<0.0001	Significant
A—pH	0.28	1	0.28	356.89	<0.0001	
B—Sucrose	0.29	1	0.29	367.62	<0.0001	
C—Peptone	1.31	1	1.31	1,674.89	<0.0001	
AB	0.021	1	0.021	26.87	0.0066	
AC	0.14	1	0.14	181.94	0.0002	
BC	0.62	1	0.62	786.43	<0.0001	
A^2^	0.34	1	0.34	438.59	<0.0001	
B^2^	1.20	1	1.20	1,538.11	<0.0001	
C^2^	0.045	1	0.045	56.93	0.0017	
A^2^B	0.60	1	0.60	772.88	<0.0001	
A^2^C	1.73	1	1.73	2,209.06	<0.0001	
AB^2^	0.24	1	0.24	305.89	<0.0001	
Residual	3.130E−003	4	7.824E−004			
Cor Total	9.16	16				

*Degree of freedom.

**Figure 7:**
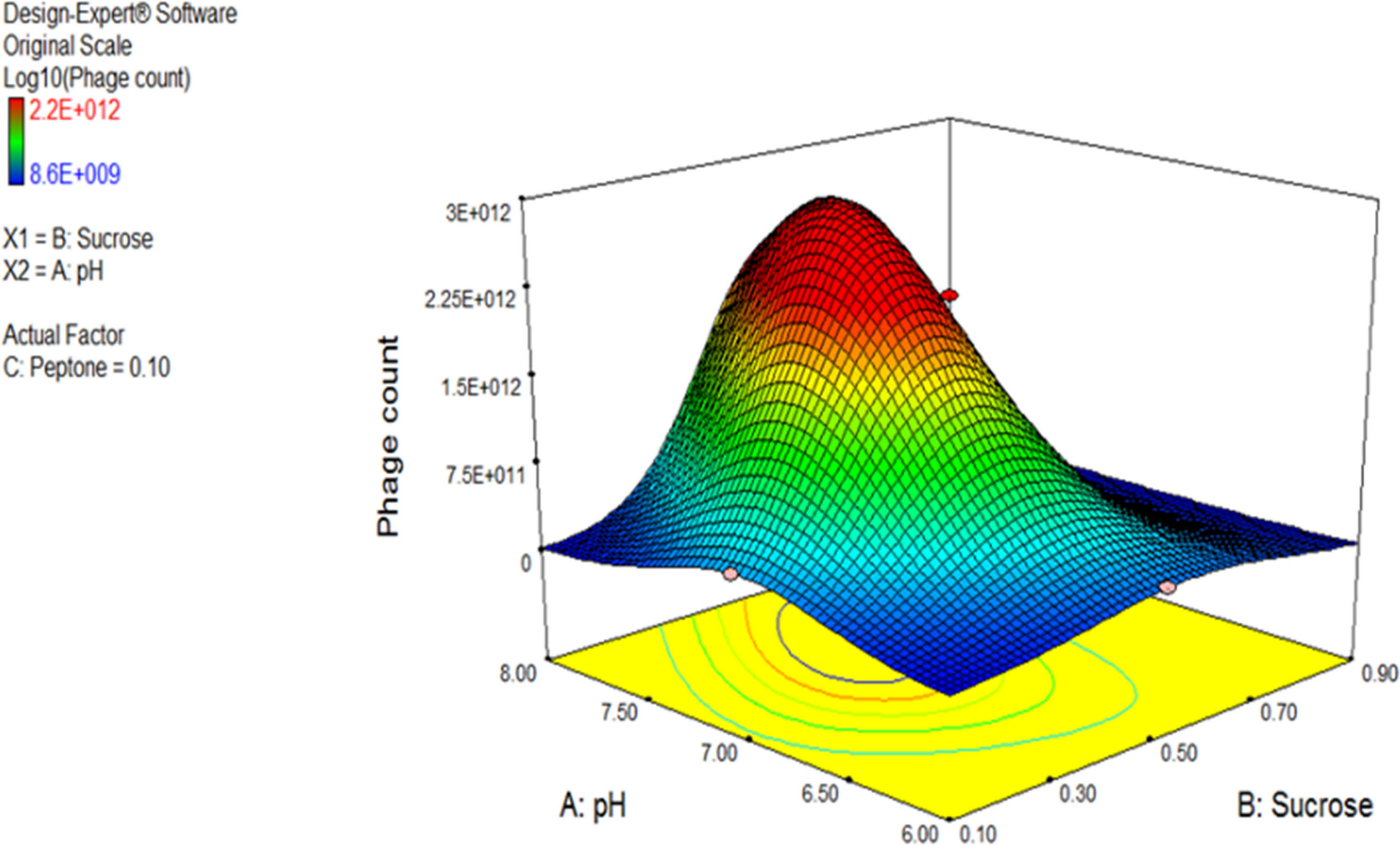
Three-dimensional (3D) response surface plot representing the effect of three parameters on bacteriophage production. pH and sucrose were plotted, and peptone was set at central level. The plots were obtained from Design Expert software^®^ v. 7.0.

The 3D response surface plot generated by Design Expert^®^ for the model F2 is provided in **Figure 7** as a graphical illustration of the interactions between the influential variables (El-Sayed et al., 2020a).

Using the numerical optimization function in the Design Expert^®^ software along with the 3D plots, the optimum conditions suggested for maximum phage production were pH 7, sucrose of 0.5% w/v, and peptone of 0.1% w/v.

##### 3.4.2.3 Model Diagnostics

**Figure 8:**
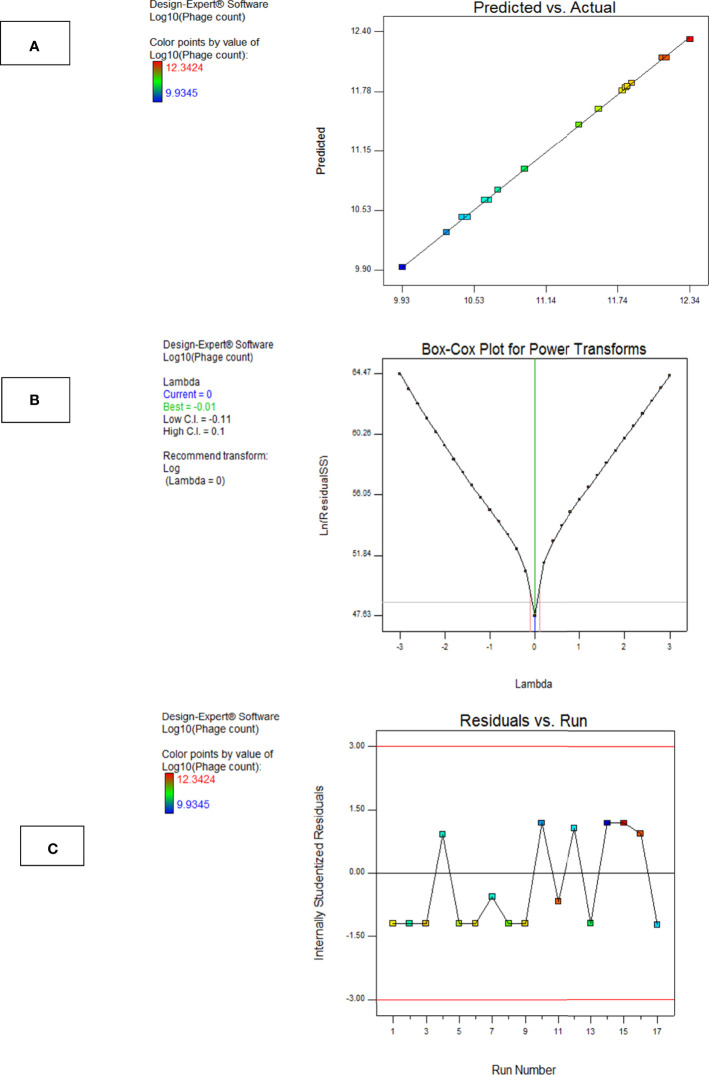
Model diagnostic plots for Model F2. **(A)** Actual vs. predicted plot, **(B)** Box–Cox plot, and **(C)** residuals versus run order plot. The plots were obtained from Design Expert software ^®^ v. 7.0.

Model diagnostics seek the assessment of a model’s validity and are presented in the form of graphical summaries as follows:

a. The Box–Cox Plot
This plot helps to determine the most appropriate power transformation to be carried out. A transformation to the base 10 log was recommended in case of phage F2 (**Figure 8A**).
b. Predicted vs. Actual Plot
The central line in this graph represents the ideal case where actual and predicted data are perfectly matching. In our case, there was a fair agreement between the actual and the predicted responses as revealed by the close distribution around the line (**Figure 8B**).
c. Residuals vs. Run Order Plot
This is a scatter plot with the residuals presented on the y-axis and the run order on the x-axis. The model F2 showed a well-behaved residual vs. run order plot where the residuals bounced arbitrarily around the residual = 0 line, suggesting model validity (**Figure 8C**).

##### 3.4.2.4. Model Experimental Verification

Phage F2 production using the suggested optimal levels of the three factors (pH 7, 0.5% w/v sucrose, and 0.1% w/v peptone) resulted in a phage titer of 2.2 × 10^12^ PFU/ml, and this value was very close to the one predicted by the model. The optimal conditions reached brought about a 2-log fold increase in phage titer as compared to that obtained under the basic unoptimized conditions (3 × 10^10^ PFU/ml).

## 4 Discussion

The exacerbation of the bacterial resistance to most of the clinically used antibiotics has prompted the readdressing of bacteriophage therapy as a promising recourse. Indeed, several sporadic risings of antibacterial phage therapy have been reported along the past century (Abedon et al., 2017; Jennes et al., 2017; Nir-Paz et al., 2019). Lytic phages are comparable to antibiotics in terms of their recognizable antibacterial effect. Moreover, there are some advantages that distinguish phage therapy, such as specific reproduction at the infection site, the merit of single or infrequent administration, and of course the privilege of being active against the otherwise pan-drug-resistant bacteria. In an attempt to isolate phages active against resistant organisms, MRSA, an example of perturbing multidrug-resistant organism, was chosen as the bacterial host for this purpose (Berryhill et al., 2021; Gordillo Altamirano and Barr, 2021). Twenty-three MRSA isolates were collected from various clinical specimens. They were resistant to nearly all the antibiotics tested except for linezolid and vancomycin. Bacteriophage isolation was carried out by incubating an environmental sample with the freshly grown bacterial host, followed by removal of bacterial cells using centrifugation and chloroform treatment. Eighty-eight environmental samples varying widely in their sources were collected for this purpose: sewage, wastewater, hospital wards and laboratory water, raw milk, the rinse of raw chicken eggs, poultry wastes, food restaurant run-offs, and the rinses of the raw meat of beef, chicken, and fish. Only five lysates continued to show positive results over a long period of time with raw chicken and fish rinses and raw milk appearing to be potential reservoirs for separation of staphylococcal lytic phages (Hyman, 2019). Hitherto, most of *S. aureus*-specific phages were reported to be separated from sewage (Han et al., 2013; Li and Zhang, 2014; Abatángelo et al., 2017; Melo et al., 2018) and milk (García et al., 2009; Kwiatek et al., 2012). To the best of our knowledge, only a few studies have described the recovery of *S. aureus* phages from chicken and fish (Duc et al., 2020). Plaque assay provides guidance on a particular lysate’s purity and the counts of viruses present as well (Anderson et al., 2011). If it results in only one form of plaques of similar morphology, size, and shape, this indicates the presence of a single pure virus. On the contrary, if it gives rise to numerous plaque forms of different characteristics, then this suggests the presence of multiple viruses. In addition, the numbers of similar plaques are inserted into a mathematical equation that approximately infers the quantity of the virus present in the original lysate in plaque forming units per ml (PFU/ml) (Anderson et al., 2011; Hyman, 2019). The initial titers of the five phage suspensions were reasonably high (>10^8^ PFU/ml) and were fairly reproducible. The lysate F2 showed only one plaque form, suggesting the presence of single virus. Plaque morphology could point to the type of a phage. Clear transparent plaques are usually characteristic of lytic (virulent) phages, whereas phages with lysogenic ability (temperate phages) feature opaque, turbid ones (Jurczak-Kurek et al., 2016; Park et al., 2020). Moreover, some phages produce plaques surrounded with halos. Halos are ascribed to the diffusion of bacteriophage enzymes that are mostly active against the cell wall of bacterial cells and the biofilms formed by some (Dakheel et al., 2019; Vukotic et al., 2020; Tan et al., 2021). Observing F2 plaques, they were clear, circular with regular entire margins, and small in diameter (3–5 mm), suggesting it being lytic (Duc et al., 2020). In addition, the plaques displayed halos around them that were observed to extend in size with increasing incubation time or when kept at room temperature for multiple days, implying an anti-biofilm activity; however, this requires further testing against biofilm-forming organisms (Dakheel et al., 2019; Vukotic et al., 2020).

Customarily, phage candidates for therapy are selected based on strict criteria; for example, being obligately lytic, possessing a broad host range, and, naturally, high stability (Klumpp and Loessner, 2013). Therefore, it was necessary to investigate some of the main characteristics of the obtained phage. The host range of a bacteriophage is bound to genus, species, and strains of bacteria that it is capable of infecting (Hyman, 2019). This, obviously, is particularly important when applying phage in therapy. A host range of a certain phage limited to a single species is preferable, as it inhibits the phage from attacking bacteria other than the one causing the disease, leaving the host’s microbiome undisturbed. Thus, in terms of species, a phage with a narrow host range is desirable. However, within this species, a phage infecting many, if not all strains, is advantageous. It insinuates its aptness for empirical application in analogy to broad-spectrum antibiotics (Hyman, 2019). The interaction between bacteriophage and host cell receptors is responsible for the phage specificity and, consequently its range of activity (Ross et al., 2016; de Jonge et al., 2019). In addition, the phage isolation procedure in the classical fashion, which happens to appoint only one host on which phages are intended to grow, might be a contributor to their normally confined host range (Hyman, 2019). As shown in the results, the five lysates proved to be broadly active, with F2, showing a polyvalent behavior, being effectual in lysis of the whole set of 22 MRSA isolates. Hence, it was selected for further studies.

The morphology of phage particles is customarily used for their prelusive classification. Even though various morphologies of bacteriophages have been discovered, and in spite of the recent proposals to abolish the traditional classification of having only one bacteriophage order (Turner et al., 2021), to this point in time, the order Caudovirales is the one comprising tailed phage families, most popularly Myoviridae, Siphoviridae, and Podoviridae. Phages of these families have an icosahedral capsid with a genome of double-stranded (ds) DNA, they but differ in their tail shape. Siphoviridae, from Greek *siphon*, meaning “tube,” is characterized by non-contractile, thin, long tails; they are often flexible and are built of stacked discs of six subunits (King et al., 2012; Othman et al., 2015; Alič et al., 2017). Negative staining and transmission electron microscopy (TEM) inspection is the most important technique used for visualization of phages (Ackermann, 2012). F2 phage suspension was introduced for TEM (Clokie and Kropinski, 2009; Kalatzis et al., 2016). The morphological features of the virus particle illustrated the following: it appeared to be tailed; therefore, it could be placed under the order Caudovirales. It had an icosahedral head with no connectors at the head-to-tail junction. It lacked base plates as well, excluding the probability of belonging to Myoviridae (Han et al., 2013; Alič et al., 2017). It was marked with a long, tubular tail (100 nm) and a head of about 34 nm in diameter. Based on the morphological similarity with the descriptions shown on Viral Zone website (“Siphoviridae ~ ViralZone,”), and according to the guidelines compiled by the International Committee on Taxonomy of Viruses (ICTV)—ninth report (King et al., 2011), it was suggested that F2 probably belongs to Siphoviridae. Even so, genome sequencing is the technique reliable when it comes to confirmation of classification and life cycle of phages. The suggested classification of the isolated phage was consistent with those demonstrated in some former studies that had managed to isolate *S. aureus Sipho*-viruses (Alič et al., 2017; Zhang et al., 2017; Cha et al., 2019; Kornienko et al., 2020). Some studies have described that the vast majority of *S. aureus* phages are members of Siphoviridae (Deghorain and Van Melderen, 2012; Klumpp and Loessner, 2013; Duc et al., 2020).

The longevity of F2 has been checked as well. It perpetuated a strong lytic effect when kept at different temperatures for 90 days.

Physicochemical stability of bacteriophages is critical for large-scale isolation, different stages of formulation and storage, and eventually real-life applications (Abdelsattar et al., 2019; Abdallah et al., 2021). The phage infectivity is influenced by numerous factors, like temperature, pH, irradiation, and the presence of certain chemicals (Mahmoud et al., 2020; Abdallah et al., 2021). Based on this, F2 was challenged by various physicochemical conditions. It appeared to possess some properties that can set it out for therapeutic purposes.

Thermal inactivation point was 60°;C after 1 h of exposure, indicating its promising suitability for storage at shelf and tolerability for hot weather in general. This observation was consistent with the thermal stability investigation similarly done by González et al. They concluded that the phage tested lost infectivity when exposed to a temperature of 60°;C for 90 min (González-Menéndez et al., 2018b). Moreover, this degree of thermal stability surpasses that of some well-known *S. aureus* bacteriophages mentioned in previous studies like SPW (Li and Zhang, 2014), SAJK-IND, and MSP (Ganaie et al., 2018), which totally lost their lytic activity above 50°;C. Nonetheless, other studies have shown higher thermal inactivation points. For instance, inactivation at 65°;C was observed by Wang et al. (2016), and the isolated phage vB_SauM_CP9 retained infectivity till 70°;C in another study by Abdallah et al. (2021). Exceptional stability up to 85°;C was also noticed by Mahmoud et al. while examining their *S. aureus* phages (Mahmoud et al., 2020).

Over the pH spectrum, F2 displayed an oscillating pattern. It was completely inactivated at the acidic end of the spectrum (pH 1) and showed a very weak lytic activity at pH 2, exhibited as a partially clear spot. Along the pH range, 3–11, it retained its activity as illustrated by totally clear spots. However, it started to decline again after exposure to the alkaline extreme (pH 12 and 13). This pattern is similar to that mentioned in a study published in 2017 (Zhang et al., 2017), as the isolated *Sipho-*virus was stable all over the pH range of 3–12. In another study performed by Abdallah et al., the bacteriophage vB_SauM_CP9 was inactivated outside the pH range of 4–9 (Abdallah et al., 2021). In a different study by Wan et al., a *S. aureus* phage was stable over a narrower range, only at pH 6, 7, and 8 (Wan Nurhafizah et al., 2017). Thus, it can be deduced that F2 phage is characterized by high stability over a very wide pH range.

Because of UV efficacy, it is becoming a common method for disinfection of water and surfaces in hospital and operation rooms (Rodriguez et al., 2014), and it has the potential to be included in phage industry processes in the future. Nevertheless, the ability of UV to induce genomic damage, genetic mutations, and recombination is well-recognized. Accordingly, UV was reported to be one of the most damaging methods of phages in very short times (Kim et al., 2018). On grounds of this theory, the UV effect on phage F2 was observed. Indeed, it could maintain its lytic ability for only 30 min of exposure to UV; its activity then weakened till it completely disappeared after 1 h, indicating that UV has a certain deadly effect on it. This finding was in accordance with the observation of some studies (Rodriguez et al., 2014; Kim et al., 2018; Hussein et al., 2019).

Upon examining the viricidal effect of some of the commonly used organic solvents (ethanol, isopropanol, and chloroform) on the phage particles, they appeared to have poor impact in killing them. Phage F2 retained its activity along most of the concentrations used; however, it suffered from a little decrease in its lytic effect starting from 50% v/v ethanol upwards. This notably agrees with the results published in 2018 by Kim et al. (2018).

If antibacterial phage therapy is to be applied publicly, the central point will be isolation of bacteriophages effective in lysis of the resistant pathogens and their mass production so as to meet the future demands. Phage propagation is highly dependent on the physiological nature of the bacterial host, especially its multiplication rate (Hadas et al., 1997; Kick et al., 2017; Nabergoj et al., 2018). Lysogenic phages unstoppably replicate in accordance with their infected hosts growth rate. Contrarily, the reproduction cycle of lytic phages, the ones most important in therapy, ends with the lysis of their host cells. The phage production process is usually sensitive to even the slightest variations in culture conditions, physiology of the organisms, and culture media composition (Egli, 2015; Nguyen et al., 2021); besides, the lytic phages run out of their production factories, with no more living bacterial cells that could be infected. Thus, for a higher phage yield, handling the phage–host interaction, and elongating the period over which bacterial cells are alive in the co-culture, could be a potential strategy when considering scaling up of phage production. Based on this, the final step of the study was designed to optimize the production of phage F2 on laboratory scale (Grieco et al., 2012; González-Menéndez et al., 2018b; Kim et al., 2021).

Production optimization is ordinarily accomplished through different approaches, some of which are conventional, like the one-factor-at-a-time (OFAT) method, where the effect of some factors influencing the production is studied individually. Nonetheless, besides being tedious and time consuming, the traditional optimization methods are not efficient in capturing the combined effect of multiple factors; therefore, OFAT approach alone is not sufficient to have a complete understanding of the bacterium-phage system behavior (Latha et al., 2017). On the contrary, other methods are more advanced such as the RSM, which is concerned with the combinatorial interaction of the factors; therefore, it is apt for studying processes where the response is affected by a blend of input variables. It is also time and cost saving. Currently, it is performed with the aid of design software. Thus, taking it on is an edge. In the present study, both approaches, OFAT and RSM, were adopted in a trial to identify the conditions conducive to enhance the bacteriophage-produced titer. Knowing that carbon and nitrogen sources are often imperative media components, two different sources were tried for each. Sucrose and peptone were the preferable sources for phage F2. This was in partial agreement with the study carried out in 2021 by Kim et al., on the staphylococcal *Kayvirus* psa-3, as they declared glycerol and glycine as the preferable sources (Kim et al., 2021). Unexpectedly, the bacteriophage F2 inclined towards the diluted side of 10^7^ CFU/ml as the preferable inoculum size that led to maximum production. This was close to the result of Kim et al., as they got 10^8^ CFU/ml as the one giving the highest phage yield (Kim et al., 2021). Since temperature is one of the most influential culture conditions, three levels of temperatures were tested, and 28°;C was the temperature that led to maximum phage count. This differed from the results reached by Gonzalez et al., as they found 38°;C to be the optimal temperature for their staphylococcal Myovirus production (González-Menéndez et al., 2018a). Next, RSM was employed, and the parameters included for investigation were pH, concentration of the selected carbon source, and concentration of the selected nitrogen source as well, each at three different levels. Using the statistical software package Design Expert^®^ v. 7.0, the D-optimal design was selected, as it provides a sensible selection of an optimal set of runs from a larger multitude of potential experiments. This design was also used in a previous similar study (González-Menéndez et al., 2018a). According to the experimental design, 17 experiments were performed in order to investigate the effect of the stated factors simultaneously, and a response surface reduced cubic model was generated.

ANOVA was used afterwards to determine the model significance and validity (Bhaumik et al., 2013). The obtained F-value was 975.10 (p-value <0.0001), which proves the significance of the model. The coefficient of determination R^2^ denotes the ability of the model to predict the variation in response. It ranges from 0 to 1 (Chen et al., 2009; El-Sayed et al., 2020a), where 1 indicates that 100% of the variation could be explained by a model. The R^2^ value derived (R^2^ = 0.97) corroborates that the developed model could explain more than 90% of the variability in response. The adjusted R^2^ (Adj R^2^)—a modified version of R^2^ that is adjusted for the number of predictors in a model—was recorded to be 0.9986. Moreover, the predicted R^2^ (Pred R^2^)—the one showing how adequately a model predicts responses for new observations—should be in reasonable agreement with Adj R^2^ and should not differ from it by more than 0.2 (El-Sayed et al., 2020b). This, too, holds true as it equals 0.9504. Adequate precision is the signal-to-noise ratio. Ratios >4 are advantageous, as it indicates appropriate model discrimination (Abdel-Hafez et al., 2014). Its value was found to be 97.680. Furthermore, the model came out to have low coefficient of variance (C.V.%), 0.25, revealing that the model was reliable (El-Sayed et al., 2020b; El-Sayed et al., 2020a). Model diagnostic plots obtained by the software proved the validity of the model. The factors impacting bacteriophage production were discerned through the p-value. The parameters, namely, A, B, and C, along with the interactions, AB, AC, BC, A^2^, B^2^, C^2^, A^2^B, A^2^C, and AB^2^, all had p-values <0.05; thus, they were significant model terms and are assumed to substantially influence phage production. This indicates that all three factors (pH, sucrose concentration, and peptone concentration) significantly influenced phage titer. Interaction is when the effect of one factor depends on the level of another factor. All interactions between the factors had significant effect on the phage production. Viewing the graphical 3D plot presentation of the model, an estimation of the ideal combination of variables for maximum response was obtained. This, together with the numerical optimization function rendered by the software, pointed to the optimum conditions for maximum phage titer. The optimum conditions were pH 7, sucrose of 0.5% w/v, and peptone of 0.1% w/v. Upon experimental verification, these conditions resulted in a phage titer of 2.2 × 10^12^ PFU/ml, and this value was very close to the one predicted by the model. Ultimately, it can be said that the optimization process managed to improve the yield of the bacteriophage F2 by 2 log-fold as compared to the basic unoptimized conditions.

## Conclusion

The constant expansion of drug-resistant bacteria has prompted the search for efficient antibacterial alternatives like phage therapy. In this study, a bacteriophage isolated from raw fish managed to effectively lyse 23 MRSA strains. This phage is suggested to belong to order Caudovirales, family Siphoviridae; however, it needs to be further characterized so as to confirm the identification. Overall, it had good stability towards some extreme conditions like temperature, pH, and UV irradiation, making it a good candidate for *in vivo* testing. In addition, optimizing the production of this phage using OFAT and RSM led to a distinctive increase in the produced titer compared to that in the unoptimized propagation conditions. In conclusion, to gain the benefits from bacteriophages as competent antibacterial therapeutic tool, primarily effective phages must go through numerous testing and well-designed clinical trials.

## Data Availability Statement

The raw data supporting the conclusions of this article will be made available by the authors, without undue reservation.

## Author Contributions

Conceived and designed the experiments: IA-A, GE-H, SE-M, and KA. Carried out the experiments: IA-A. Carried out the statistical analysis: GE-H. Drafting the paper: IA-A, GE-H, SE-M, MA, and KA. Wrote the paper in its final format: GE-H, MA, and NH. All authors contributed to the article and approved the submitted version.

## Conflict of Interest

The authors declare that the research was conducted in the absence of any commercial or financial relationships that could be construed as a potential conflict of interest.

## Publisher’s Note

All claims expressed in this article are solely those of the authors and do not necessarily represent those of their affiliated organizations, or those of the publisher, the editors and the reviewers. Any product that may be evaluated in this article, or claim that may be made by its manufacturer, is not guaranteed or endorsed by the publisher.

## References

[B1] Abatángelo V. Bacci N. P. Boncompain C. A. Amadio A. A. Carrasco S. Suárez C. A.. (2017). Broad-Range Lytic Bacteriophages That Kill Staphylococcus Aureus Local Field Strains. PLoS One 12, e0181671. doi: 10.1371/journal.pone.0181671 28742812 PMC5526547

[B2] Abdallah K. Tharwat A. Gharieb R. (2021). High Efficacy of a Characterized Lytic Bacteriophage in Combination With Thyme Essential Oil Against Multidrug-Resistant Staphylococcus Aureus in Chicken Products. Iran. J. Vet. Res. 22, 24–32. doi: 10.22099/ijvr.2020.38083.5543 34149853 PMC8195302

[B3] Abdel-Hafez S. M. Hathout R. M. Sammour O. A. (2014). Towards Better Modeling of Chitosan Nanoparticles Production: Screening Different Factors and Comparing Two Experimental Designs. Int. J. Biol. Macromol. 64, 334–340. doi: 10.1016/j.ijbiomac.2013.11.041 24355618

[B4] Abdelsattar A. S. Abdelrahman F. Dawoud A. Connerton I. F. El-Shibiny A. (2019). Encapsulation of E. Coli Phage ZCEC5 in Chitosan–Alginate Beads as a Delivery System in Phage Therapy. AMB Exp. 9, 1–9. doi: 10.1186/s13568-019-0810-9 PMC6579803 31209685

[B5] Abedon S. T. García P. Mullany P. Aminov R. (2017). Editorial: Phage Therapy: Past, Present and Future. Front. Microbiol. 8. doi: 10.3389/fmicb.2017.00981 PMC5471325 28663740

[B6] Ackermann H.-W. (1998). Tailed Bacteriophages: The Order Caudovirales. Adv. Virus Res. 51, 135–201. doi: 10.1016/S0065-3527(08)60785-X 9891587 PMC7173057

[B7] Ackermann H. W. (2012). “Bacteriophage Electron Microscopy,” in Advances in Virus Research (Quebec, Canada: Elsevier), 1–32. doi: 10.1016/B978-0-12-394621-8.00017-0 22420849

[B8] Ackermann H. (2012). Bacteriophages, Part a Vol. 82 (Academic Press), 1–32. doi: 10.1016/B978-0-12-394621-8.00017-0

[B9] Ács N. Gambino M. Brøndsted L. (2020). Bacteriophage Enumeration and Detection Methods. Front. Microbiol. 11. doi: 10.3389/fmicb.2020.594868 PMC7644846 33193274

[B10] Adams M. H. (1959). Bacteriophages (New York, USA: Interscience Publishers).

[B11] Algammal A. M. Hetta H. F. Elkelish A. Alkhalifah D. H. H. Hozzein W. N. Batiha G. E.-S.. (2020). Methicillin-Resistant Staphylococcus Aureus (MRSA): One Health Perspective Approach to the Bacterium Epidemiology, Virulence Factors, Antibiotic-Resistance, and Zoonotic Impact. Infect. Drug Resist. 13, 3255–3265. doi: 10.2147/IDR.S272733 33061472 PMC7519829

[B12] Alič Š. Naglič T. Tušek-Žnidarič M. Ravnikar M. Rački N. Peterka M.. (2017). Newly Isolated Bacteriophages From the Podoviridae, Siphoviridae, and Myoviridae Families Have Variable Effects on Putative Novel Dickeya Spp. Front. Microbiol. 0. doi: 10.3389/fmicb.2017.01870 PMC5626979 29033917

[B13] Anderson B. Rashid M. H. Carter C. Pasternack G. Rajanna C. Revazishvili T.. (2011). Enumeration of Bacteriophage Particles. Bacteriophage 1, 86–93. doi: 10.4161/bact.1.2.15456 22334864 PMC3278645

[B14] Berryhill B. A. Huseby D. L. McCall I. C. Hughes D. Levin B. R. (2021). Evaluating the Potential Efficacy and Limitations of a Phage for Joint Antibiotic and Phage Therapy of *Staphylococcus Aureus* Infections. Proc. Natl. Acad. Sci. 118, e2008007118. doi: 10.1073/pnas.2008007118 33649203 PMC7958385

[B15] Bhaumik R. Mondal N. K. Chattoraj S. Datta J. K. (2013). Application of Response Surface Methodology for Optimization of Fluoride Removal Mechanism by Newly Developed Biomaterial. Am. J. Anal. Chem. 4, 404–419. doi: 10.4236/ajac.2013.48051

[B16] Brown D. F. J. Edwards D. I. Hawkey P. M. Morrison D. Ridgway G. L. Towner K. J.. (2005). Guidelines for the Laboratory Diagnosis and Susceptibility Testing of Methicillin-Resistant Staphylococcus Aureus (MRSA). J. Antimicrob. Chemother. 56, 1000–1018. doi: 10.1093/jac/dki372 16293678

[B17] Cha Y. Chun J. Son B. Ryu S. (2019). Characterization and Genome Analysis of Staphylococcus Aureus Podovirus CSA13 and Its Anti-Biofilm Capacity. Viruses 11, 54. doi: 10.3390/v11010054 PMC6356323 30642091

[B18] Chen X.-C. Bai J.-X. Cao J.-M. Li Z.-J. Xiong J. Zhang L.. (2009). Medium Optimization for the Production of Cyclic Adenosine 3′,5′-Monophosphate by Microbacterium Sp. No. 205 Using Response Surface Methodology. Bioresour. Technol. 100, 919–924. doi: 10.1016/j.biortech.2008.07.062 18778935

[B19] Clarke P. H. Cowan S. T. (1952). Biochemical Methods for Bacteriology. J. Gen. Microbiol. 6, 187–197. doi: 10.1099/00221287-6-1-2-187 14927866

[B20] CLSI in 2018. Available at: https://clsi.org/2018/ (Accessed December 17, 2021).

[B21] Clokie M. R. J. Kropinski A. (Eds.) (2009). “Bacteriophages,” in Methods and Protocols, Volume 1: Isolation, Characterization, and Interactions (New Jersey, USA: Humana Press). doi: 10.1007/978-1-60327-164-6

[B22] Craft K. M. Nguyen J. M. Berg L. J. Townsend S. D. (2019). Methicillin-Resistant Staphylococcus Aureus (MRSA): Antibiotic-Resistance and the Biofilm Phenotype. MedChemComm 10, 1231–1241. doi: 10.1039/c9md00044e 31534648 PMC6748282

[B23] Dakheel K. H. Rahim R. A. Neela V. K. Al-Obaidi J. R. Hun T. G. Isa M. N. M.. (2019). Genomic Analyses of Two Novel Biofilm-Degrading Methicillin-Resistant Staphylococcus Aureus Phages. BMC Microbiol. 19, 114. doi: 10.1186/s12866-019-1484-9 31138130 PMC6540549

[B24] Deghorain M. Van Melderen L. (2012). The Staphylococci Phages Family: An Overview. Viruses 4, 3316–3335. doi: 10.3390/v4123316 23342361 PMC3528268

[B25] de Jonge P. A. Nobrega F. L. Brouns S. J. J. Dutilh B. E. (2019). Molecular and Evolutionary Determinants of Bacteriophage Host Range. Trends Microbiol. 27, 51–63. doi: 10.1016/j.tim.2018.08.006 30181062

[B26] D’Herelle F. (1929). The Bacteriophage and Its Behavior. J. Immunol. 4, 1–8.

[B27] Dodds D. R. (2017). Antibiotic Resistance: A Current Epilogue. Biochem. Pharmacol. 134, 139–146. doi: 10.1016/j.bcp.2016.12.005 27956111

[B28] Duc H. M. Son H. M. Ngan P. H. Sato J. Masuda Y. Honjoh K.. (2020). Isolation and Application of Bacteriophages Alone or in Combination With Nisin Against Planktonic and Biofilm Cells of Staphylococcus Aureus. Appl. Microbiol. Biotechnol. 104, 5145–5158. doi: 10.1007/s00253-020-10581-4 32248441

[B29] Egli T. (2015). Microbial Growth and Physiology: A Call for Better Craftsmanship. Front. Microbiol. 6. doi: 10.3389/fmicb.2015.00287 PMC4396425 25926822

[B30] El-Dougdoug N. Nasr-Eldin M. Azzam M. Mohamed A. Hazaa M. (2019). Improving Wastewater Treatment Using Dried Banana Leaves and Bacteriophage Cocktail. Egypt. J. Bot. 0, 0–0. doi: 10.21608/ejbo.2019.7597.1295

[B31] El-Housseiny G. S. Aboulwafa M. M. Aboshanab K. A. Hassouna N. A. H. (2016). Optimization of Rhamnolipid Production by P. Aeruginosa Isolate P6. J. Surfact. Deterg. 19, 943–955. doi: 10.1007/s11743-016-1845-4

[B32] El-Housseiny G. Shams G. Ghobashi Z. Mamdouh R. Almaqsod I. Saleh S. (2021). Optimization of Antifungal Activity by Bacillus Subtilis Isolate CCASU 2021-4 Using Response Surface Methodology. Arch. Pharm. Sci. Ain. Shams. Univ. 5, 171–183. doi: 10.21608/aps.2021.80383.1063

[B33] El-Sayed S. E. Abdelaziz N. A. El-Housseiny G. S. Aboshanab K. M. (2020a). Octadecyl 3-(3, 5-Di-Tert-Butyl-4-Hydroxyphenyl) Propanoate, an Antifungal Metabolite of Alcaligenes Faecalis Strain MT332429 Optimized Through Response Surface Methodology. Appl. Microbiol. Biotechnol. 104, 10755–10768. doi: 10.1007/s00253-020-10962-9 33090249

[B34] El-Sayed S. E. El-Housseiny G. S. Abdelaziz N. A. El-Ansary M. R. Aboshanab K. M. (2020b). Optimized Production of the Allylamine Antifungal &Ldquo;Terbinafine&rdquo; by Lysinibacillus Isolate MK212927 Using Response Surface Methodology. Infect. Drug Resist. 13, 3613–3626. doi: 10.2147/IDR.S267590 33116681 PMC7571585

[B35] Erol H. B. Kaskatepe B. Bakkaloglu Z. Suzuk Yildiz S. (2021). The Evaluation of Five Commercial Bacteriophage Cocktails Against Methicillin-Resistant Staphylococcus Aureus Isolated From Nasal Swab Samples. Arch. Microbiol. 203, 5735–5743. doi: 10.1007/s00203-021-02564-4 34487189

[B36] Felten A. Grandry B. Lagrange P. H. Casin I. (2002). Evaluation of Three Techniques for Detection of Low-Level Methicillin-Resistant Staphylococcus Aureus (MRSA): A Disk Diffusion Method With Cefoxitin and Moxalactam, the Vitek 2 System, and the MRSA-Screen Latex Agglutination Test. J. Clin. Microbiol. 40, 2766–2771. doi: 10.1128/JCM.40.8.2766-2771.2002 12149327 PMC120619

[B37] Ferraro M. J. (2000) Performance Standards for Antimicrobial Disk Susceptibility Tests (NCCLS). Available at: https://scholar.google.com/scholar_lookup?title=Performance+standards+for+antimicrobial+disk+susceptibility+tests&author=Ferraro%2C+Mary+Jane.&publication_year=2000 (Accessed September 9, 2021).

[B38] Fisk A. (1940). The Technique of the Coagulase Test for Staphylococci. Br. J. Exp. Pathol. 21 (5), 311–314.

[B39] Fortier L.-C. Moineau S. (2009). “Phage Production and Maintenance of Stocks, Including Expected Stock Lifetimes,” in Bacteriophages: Methods and Protocols, Volume 1: Isolation, Characterization, and Interactions Methods in Molecular BiologyTM. Eds. Clokie M. R. J. Kropinski A. M. (Humana Press: Totowa, NJ), 203–219. doi: 10.1007/978-1-60327-164-6_19 19066823

[B40] Ganaie M. Y. Qureshi S. Kashoo Z. Wani S. A. Hussain M. I. Kumar R.. (2018). Isolation and Characterization of Two Lytic Bacteriophages Against Staphylococcus Aureus From India: Newer Therapeutic Agents Against Bovine Mastitis. Vet. Res. Commun. 42, 289–295. doi: 10.1007/s11259-018-9736-y 30219981

[B41] Gao W. Zhang L. (2021). Nanomaterials Arising Amid Antibiotic Resistance. Nat. Rev. Microbiol. 19, 5–6. doi: 10.1038/s41579-020-00469-5 33024312 PMC7538279

[B42] García P. Martínez B. Obeso J. M. Lavigne R. Lurz R. Rodríguez A. (2009). Functional Genomic Analysis of Two Staphylococcus Aureus Phages Isolated From the Dairy Environment. Appl. Environ. Microbiol. 75, 7663–7673. doi: 10.1128/AEM.01864-09 19837832 PMC2794103

[B43] Gayar M. H. E. Aboulwafa M. M. Aboshanab K. M. Hassouna N.A.h. (2014). Virulence Characters of Some Methicillin Resistant Staphylococcus Aureus Isolates. Arch. Clin. Microbiol. 5 (4), 1–14. doi: 10.3823/283

[B44] Gill J. J. Hyman P. (2010). Phage Choice, Isolation, and Preparation for Phage Therapy. Curr. Pharm. Biotechnol. 11, 2–14. doi: 10.2174/138920110790725311 20214604

[B45] González-Menéndez E. Arroyo-López F. N. Martínez B. García P. Garrido-Fernández A. Rodríguez A. (2018a). Optimizing Propagation of Staphylococcus Aureus Infecting Bacteriophage Vb_SauM-phiIPLA-RODI on Staphylococcus Xylosus Using Response Surface Methodology. Viruses 10, 153. doi: 10.3390/v10040153 PMC5923447 29584701

[B46] González-Menéndez E. Fernández L. Gutiérrez D. Pando D. Martínez B. Rodríguez A.. (2018b). Strategies to Encapsulate the Staphylococcus Aureus Bacteriophage phiIPLA-RODI. Viruses 10 (9), 495. doi: 10.3390/v10090495 PMC6163856 30217072

[B47] Gordillo Altamirano F. L. Barr J. J. (2021). Unlocking the Next Generation of Phage Therapy: The Key is in the Receptors. Curr. Opin. Biotechnol. 68, 115–123. doi: 10.1016/j.copbio.2020.10.002 33202354

[B48] Grema H. A. (2015). Methicillin Resistant Staphylococcus Aureus (MRSA): A Review. Adv. Anim. Vet. Sci. 3, 79–98. doi: 10.14737/journal.aavs/2015/3.2.79.98

[B49] Grieco S.-H. H. Wong A. Y. K. Dunbar W. S. MacGillivray R. T. A. Curtis S. B. (2012). Optimization of Fermentation Parameters in Phage Production Using Response Surface Methodology. J. Ind. Microbiol. Biotechnol. 39, 1515–1522. doi: 10.1007/s10295-012-1148-3 22714954

[B50] Hadas H. Einav M. Fishov I. Zaritsky A. (1997). Bacteriophage T4 Development Depends on the Physiology of its Host Escherichia Coli. Microbiology 143, 179–185. doi: 10.1099/00221287-143-1-179 9025292

[B51] Hallajzadeh M. Mojtahedi A. Mahabadi V. P. Amirmozafari N. (2019). Isolation and *In Vitro* Evaluation of Bacteriophage Against Methicillin-Resistant Staphylococcus Aureus (MRSA) From Burn Wounds. Arch. Clin. Microbiol. 10, 1–8. doi: 10.36648/1989-8436.10.6.98

[B52] Han J. E. Kim J. H. Hwang S. Y. Choresca C. H. Shin S. P. Jun J. W.. (2013). Isolation and Characterization of a Myoviridae Bacteriophage Against Staphylococcus Aureus Isolated From Dairy Cows With Mastitis. Res. Vet. Sci. 95, 758–763. doi: 10.1016/j.rvsc.2013.06.001 23790669

[B53] Home | AMR Review. Available at: https://amr-review.org/ (Accessed November 6, 2020).

[B54] Hubálek Z. (2003). Protectants Used in the Cryopreservation of Microorganisms. Cryobiology 46, 205–229. doi: 10.1016/s0011-2240(03)00046-4 12818211

[B55] Hussein Y. El-Masry S. Faiesal A. El-Dougdoug Kh. Othman B. (2019). PHYSICO-CHEMICAL PROPERTIES OF SOME LISTERIA PHAGES. Arab. Univ. J. Agric. Sci. 27, 175–183. doi: 10.21608/ajs.2019.43334

[B56] Hyman P. (2019). Phages for Phage Therapy: Isolation, Characterization, and Host Range Breadth. Pharmaceuticals 12, 35. doi: 10.3390/ph12010035 PMC6469166 30862020

[B57] Jamalludeen N. Johnson R. P. Friendship R. Kropinski A. M. Lingohr E. J. Gyles C. L. (2007). Isolation and Characterization of Nine Bacteriophages That Lyse O149 Enterotoxigenic Escherichia Coli. Vet. Microbiol. 124, 47–57. doi: 10.1016/j.vetmic.2007.03.028 17560053

[B58] Jennes S. Merabishvili M. Soentjens P. Pang K. W. Rose T. Keersebilck E.. (2017). Use of Bacteriophages in the Treatment of Colistin-Only-Sensitive Pseudomonas Aeruginosa Septicaemia in a Patient With Acute Kidney Injury-a Case Report. Crit. Care Lond. Engl. 21, 129. doi: 10.1186/s13054-017-1709-y PMC5460490 28583189

[B59] Jurczak-Kurek A. Gąsior T. Nejman-Faleńczyk B. Bloch S. Dydecka A. Topka G.. (2016). Biodiversity of Bacteriophages: Morphological and Biological Properties of a Large Group of Phages Isolated From Urban Sewage. Sci. Rep. 6, 34338. doi: 10.1038/srep34338 27698408 PMC5048108

[B60] Kalatzis P. G. Bastías R. Kokkari C. Katharios P. (2016). Isolation and Characterization of Two Lytic Bacteriophages, φst2 and φgrn1; Phage Therapy Application for Biological Control of Vibrio Alginolyticus in Aquaculture Live Feeds. One 11, e0151101. doi: 10.1371/journal.pone.0151101 PMC4780772 26950336

[B61] Kateete D. P. Kimani C. N. Katabazi F. A. Okeng A. Okee M. S. Nanteza A.. (2010). Identification of Staphylococcus Aureus: DNase and Mannitol Salt Agar Improve the Efficiency of the Tube Coagulase Test. Ann. Clin. Microbiol. Antimicrob. 9, 23. doi: 10.1186/1476-0711-9-23 20707914 PMC2927478

[B62] Kick B. Hensler S. Praetorius F. Dietz H. Weuster-Botz D. (2017). Specific Growth Rate and Multiplicity of Infection Affect High-Cell-Density Fermentation With Bacteriophage M13 for ssDNA Production. Biotechnol. Bioeng. 114, 777–784. doi: 10.1002/bit.26200 27748519

[B63] Kim S. Kim S.-H. Rahman M. Kim J. (2018). Characterization of a Salmonella Enteritidis Bacteriophage Showing Broad Lytic Activity Against Gram-Negative Enteric Bacteria. J. Microbiol. 56, 917–925. doi: 10.1007/s12275-018-8310-1 30361974

[B64] Kim S. G. Kwon J. Giri S. S. Yun S. Kim H. J. Kim S. W.. (2021). Strategy for Mass Production of Lytic Staphylococcus Aureus Bacteriophage pSa-3: Contribution of Multiplicity of Infection and Response Surface Methodology. Microb. Cell Factor. 20, 56, 1–12. doi: 10.1186/s12934-021-01549-8 PMC7923500 33653327

[B65] King A. M. Q. Adams M. J. Carstens E. B. Lefkowitz E. J. (Eds.) (2012). “Family - Siphoviridae,” in Virus Taxonomy (San Diego: Elsevier), 86–98. doi: 10.1016/B978-0-12-384684-6.00004-5

[B66] King A. M. Lefkowitz E. Adams M. J. Carstens E. B. (2011). “Virus Taxonomy,” in Ninth Report of the International Committee on Taxonomy of Viruses (Oxfored, UK: Elsevier).

[B67] Klumpp J. Loessner M. J. (2013). Listeria Phages. Bacteriophage 3, e26861. doi: 10.4161/bact.26861 24251077 PMC3827098

[B68] Kornienko M. Kuptsov N. Gorodnichev R. Bespiatykh D. Guliaev A. Letarova M.. (2020). Contribution of Podoviridae and Myoviridae Bacteriophages to the Effectiveness of Anti-Staphylococcal Therapeutic Cocktails. Sci. Rep. 10, 18612. doi: 10.1038/s41598-020-75637-x 33122703 PMC7596081

[B69] Kwiatek M. Parasion S. Mizak L. Gryko R. Bartoszcze M. Kocik J. (2012). Characterization of a Bacteriophage, Isolated From a Cow With Mastitis, That is Lytic Against Staphylococcus Aureus Strains. Arch. Virol. 157, 225–234. doi: 10.1007/s00705-011-1160-3 22045271

[B70] Latha S. Sivaranjani G. Dhanasekaran D. (2017). Response Surface Methodology: A non-Conventional Statistical Tool to Maximize the Throughput of Streptomyces Species Biomass and Their Bioactive Metabolites. Crit. Rev. Microbiol. 43, 567–582. doi: 10.1080/1040841X.2016.1271308 28129718

[B71] Li L. Zhang Z. (2014). Isolation and Characterization of a Virulent Bacteriophage SPW Specific for Staphylococcus Aureus Isolated From Bovine Mastitis of Lactating Dairy Cattle. Mol. Biol. Rep. 41, 5829–5838. doi: 10.1007/s11033-014-3457-2 24981924

[B72] Łubowska N. Grygorcewicz B. Kosznik-Kwaśnicka K. Zauszkiewicz-Pawlak A. Węgrzyn A. Dołęgowska B.. (2019). Characterization of the Three New Kayviruses and Their Lytic Activity Against Multidrug-Resistant Staphylococcus Aureus. Microorganisms 7, 471. doi: 10.3390/microorganisms7100471 PMC6843549 31635437

[B73] Luong T. Salabarria A.-C. Roach D. R. (2020). Phage Therapy in the Resistance Era: Where Do We Stand and Where Are We Going? Clin. Ther. 42, 1659–1680. doi: 10.1016/j.clinthera.2020.07.014 32883528

[B74] Mahmoud E. R. A. Ahmed H. A. H. Abo-senna A. S. M. Riad O. K. M. Shadi M. M. A. A.-R. A. (2020). Isolation and Characterization of Six Gamma-Irradiated Bacteriophages Specific for MRSA and VRSA Isolated From Skin Infections. J. Radiat. Res. Appl. Sci. 0, 1–10. doi: 10.1080/16878507.2020.1795564

[B75] Meek R. W. Vyas H. Piddock L. J. V. (2015). Nonmedical Uses of Antibiotics: Time to Restrict Their Use? PLoS Biol. 13, e1002266. doi: 10.1371/journal.pbio.1002266 26444324 PMC4621705

[B76] Melo L. D. R. Brandão A. Akturk E. Santos S. B. Azeredo J. (2018). Characterization of a New Staphylococcus Aureus Kayvirus Harboring a Lysin Active Against Biofilms. Viruses 10, 182. doi: 10.3390/v10040182 PMC5923476 29642449

[B77] Montassier E. Valdés-Mas R. Batard E. Zmora N. Dori-Bachash M. Suez J.. (2021). Probiotics Impact the Antibiotic Resistance Gene Reservoir Along the Human GI Tract in a Person-Specific and Antibiotic-Dependent Manner. Nat. Microbiol. 6, 1043–1054. doi: 10.1038/s41564-021-00920-0 34226711 PMC8318886

[B78] Moodley A. Kot W. Nälgård S. Jakociune D. Neve H. Hansen L. H.. (2019). Isolation and Characterization of Bacteriophages Active Against Methicillin-Resistant Staphylococcus Pseudintermedius. Res. Vet. Sci. 122, 81–85. doi: 10.1016/j.rvsc.2018.11.008 30468880

[B79] MRSA | CDC (2019). Available at: https://www.cdc.gov/mrsa/index.html (Accessed June 8, 2020).

[B80] Nabergoj D. Modic P. Podgornik A. (2018). Effect of Bacterial Growth Rate on Bacteriophage Population Growth Rate. MicrobiologyOpen 7, e00558. doi: 10.1002/mbo3.558 29195013 PMC5911998

[B81] Nasser A. Azizian R. Tabasi M. Khezerloo J. K. Heravi F. S. Kalani M. T.. (2019). Specification of Bacteriophage Isolated Against Clinical Methicillin-Resistant Staphylococcus Aureus. Osong. Public Health Res. Perspect. 10, 20–24. doi: 10.24171/j.phrp.2019.10.1.05 30847267 PMC6396822

[B82] Nataraj B. H. Mallappa R. H. (2021). Antibiotic Resistance Crisis: An Update on Antagonistic Interactions Between Probiotics and Methicillin-Resistant Staphylococcus Aureus (MRSA). Curr. Microbiol. 78, 2194–2211. doi: 10.1007/s00284-021-02442-8 33881575

[B83] Nguyen J. Fernandez V. Pontrelli S. Sauer U. Ackermann M. Stocker R. (2021). A Distinct Growth Physiology Enhances Bacterial Growth Under Rapid Nutrient Fluctuations. Nat. Commun. 12, 3662. doi: 10.1038/s41467-021-23439-8 34135315 PMC8209047

[B84] Nir-Paz R. Gelman D. Khouri A. Sisson B. M. Fackler J. Alkalay-Oren S.. (2019). Successful Treatment of Antibiotic-Resistant, Poly-Microbial Bone Infection With Bacteriophages and Antibiotics Combination. Clin. Infect. Dis. 69, 2015–2018. doi: 10.1093/cid/ciz222 30869755

[B85] O' Neill J. (2016).Tackling Drug-Resistant Infections Globally. In: Final Report and Recommendations the Review on Antimicrobial Resistance| AMR Review. Available at: https://amr-review.org/ (Accessed November 6, 2020).

[B86] Othman B. A. Askora A. Abo-Senna A. S. M. (2015). Isolation and Characterization of a Siphoviridae Phage Infecting Bacillus Megaterium From a Heavily Trafficked Holy Site in Saudi Arabia. Folia Microbiol. (Praha) 60, 289–295. doi: 10.1007/s12223-015-0375-1 25624065

[B87] Park D.-W. Lim G. Lee Y. Park J.-H. (2020). Characteristics of Lytic Phage Vb_EcoM-ECP26 and Reduction of Shiga-Toxin Producing Escherichia Coli on Produce Romaine. Appl. Biol. Chem. 63, 19. doi: 10.1186/s13765-020-00502-4

[B88] Patel J. B. Clinical and Laboratory Standards Institute (2017). Performance Standards for Antimicrobial Susceptibility Testing (Wayne, USA).

[B89] Pertics B. Z. Szénásy D. Dunai D. Born Y. Fieseler L. Kovács T.. (2020). Isolation of a Novel Lytic Bacteriophage Against a Nosocomial Methicillin-Resistant Staphylococcus Aureus Belonging to ST45. BioMed. Res. Int. 2020, e5463801. doi: 10.1155/2020/5463801 PMC7773469 33426055

[B90] Prakash O. Nimonkar Y. Shouche Y. S. (2013). Practice and Prospects of Microbial Preservation. FEMS Microbiol. Lett. 339, 1–9. doi: 10.1111/1574-6968.12034 23083094

[B91] Raina S. K. (2019). State of the Globe: Antimicrobial Resistance: Need for De-Compartmentalization of Action. J. Glob. Infect. Dis. 11, 133–134. doi: 10.4103/jgid.jgid_178_18 31849432 PMC6906889

[B92] Reuter M. Kruger D. H. (2020). Approaches to Optimize Therapeutic Bacteriophage and Bacteriophage-Derived Products to Combat Bacterial Infections. Virus Genes 56, 136–149. doi: 10.1007/s11262-020-01735-7 32036540 PMC7223754

[B93] Rodriguez R. A. Bounty S. Beck S. Chan C. McGuire C. Linden K. G. (2014). Photoreactivation of Bacteriophages After UV Disinfection: Role of Genome Structure and Impacts of UV Source. Water Res. 55, 143–149. doi: 10.1016/j.watres.2014.01.065 24607520

[B94] Ross A. Ward S. Hyman P. (2016). More Is Better: Selecting for Broad Host Range Bacteriophages. Front. Microbiol. 7. doi: 10.3389/fmicb.2016.01352 PMC5014875 27660623

[B95] Sambrook J. Fritsch E. F. Maniatis T. World Veg. Cent (1989)Molecular Cloning. In: A Laboratory Manual. Available at: https://worldveg.tind.io/record/16489 (Accessed July 30, 2021).

[B96] Santajit S. Indrawattana N. (2016). Mechanisms of Antimicrobial Resistance in ESKAPE Pathogens. BioMed. Res. Int. 2016, 2475067. doi: 10.1155/2016/2475067 27274985 PMC4871955

[B97] Serwer P. Hayes S. J. Zaman S. Lieman K. Rolando M. Hardies S. C. (2004). Improved Isolation of Undersampled Bacteriophages: Finding of Distant Terminase Genes. Virology 329, 412–424. doi: 10.1016/j.virol.2004.08.021 15518819

[B98] Shetty N. Hill G. Ridgway G. L. (1998). The Vitek Analyser for Routine Bacterial Identification and Susceptibility Testing: Protocols, Problems, and Pitfalls. J. Clin. Pathol. 51, 316–323. doi: 10.1136/jcp.51.4.316 9659247 PMC500679

[B99] Shibabaw A. Abebe T. Mihret A. (2014). Antimicrobial Susceptibility Pattern of Nasal Staphylococcus Aureus Among Dessie Referral Hospital Health Care Workers, Dessie, Northeast Ethiopia. Int. J. Infect. Dis. 25, 22–25. doi: 10.1016/j.ijid.2014.03.1386 24813590

[B100] Siddiqui A. H. Koirala J. (2020). “Methicillin Resistant Staphylococcus Aureus (MRSA). In: StatPearls (Treasure Island (FL: StatPearls Publishing). Available at: http://www.ncbi.nlm.nih.gov/books/NBK482221/ (Accessed June 8, 2020). 29489200

[B101] Siphoviridae (2011). In: An Overview | ScienceDirect Topics. Available at: https://www.sciencedirect.com/topics/agricultural-and-biological-sciences/siphoviridae (Accessed December 8, 2021).

[B102] Siphoviridae. In: ViralZone (2011). Available at: https://viralzone.expasy.org/142?outline=all_by_species (Accessed December 4, 2021).

[B103] Stenholm A. R. Dalsgaard I. Middelboe M. (2008). Isolation and Characterization of Bacteriophages Infecting the Fish Pathogen *Flavobacterium Psychrophilum* . Appl. Environ. Microbiol. 74, 4070–4078. doi: 10.1128/AEM.00428-08 18469131 PMC2446521

[B104] Tan C. W. Rukayadi Y. Hasan H. Abdul-Mutalib N.-A. Jambari N. N. Hara H.. (2021). Isolation and Characterization of Six Vibrio Parahaemolyticus Lytic Bacteriophages From Seafood Samples. Front. Microbiol. 12. doi: 10.3389/fmicb.2021.616548 PMC7987779 33776954

[B105] Turner D. Kropinski A. M. Adriaenssens E. M. (2021). A Roadmap for Genome-Based Phage Taxonomy. Viruses 13, 506. doi: 10.3390/v13030506 33803862 PMC8003253

[B106] Vahedi A. Soltan Dallal M. M. Douraghi M. Nikkhahi F. Rajabi Z. Yousefi M.. (2018). Isolation and Identification of Specific Bacteriophage Against Enteropathogenic Escherichia Coli (EPEC) and *In Vitro* and *In Vivo* Characterization of Bacteriophage. FEMS Microbiol. Lett. 365 (16), 1–6. doi: 10.1093/femsle/fny136 29945166

[B107] Vukotic G. Obradovic M. Novovic K. Di Luca M. Jovcic B. Fira D.. (2020). Characterization, Antibiofilm, and Depolymerizing Activity of Two Phages Active on Carbapenem-Resistant Acinetobacter Baumannii. Front. Med. 7. doi: 10.3389/fmed.2020.00426 PMC7461965 32974360

[B108] Waglechner N. Wright G. D. (2017). Antibiotic Resistance: It’s Bad, But Why Isn’t it Worse? BMC Biol. 15, 84. doi: 10.1186/s12915-017-0423-1 28915805 PMC5603022

[B109] Wang Z. Zheng P. Ji W. Fu Q. Wang H. Yan Y.. (2016). SLPW: A Virulent Bacteriophage Targeting Methicillin-Resistant Staphylococcus Aureus *In Vitro* and *In Vivo* . Front. Microbiol. 7. doi: 10.3389/fmicb.2016.00934 PMC4908117 27379064

[B110] Wan Nurhafizah W. I. Aznan A. S. Saari N. A. LeeKok L. Musa N. Razzak L. A.. (2017). *In-Vitro* Characterization of Lytic Bacteriophage PhVh6 as Potential Biocontrol Agent Against Pathogenic Vibrio Harveyi. AACL Bioflux. 10, 64–76. Available at: http://www.bioflux.com.ro/docs/2017.64-76.pdf (accessed on 7th May, 2022).

[B111] Weinstein M. P. (2018). M100-Performance Standards for Antimicrobial Susceptibility Testing. 28th edition (New Jersey, US: Cinical and Lboratory).

[B112] Wencewicz T. A. (2019). Crossroads of Antibiotic Resistance and Biosynthesis. J. Mol. Biol. 431, 3370–3399. doi: 10.1016/j.jmb.2019.06.033 31288031 PMC6724535

[B113] WHO (2017). https://www.who.int/news/item/27-02-2017-who-publishes-list-of-bacteria-for-which-new-antibiotics-are-urgently-needed [Accessed March 22, 2022].

[B114] Wommack K. E. Williamson K. E. Helton R. R. Bench S. R. Winget D. M. (2009). “Methods for the Isolation of Viruses From Environmental Samples in Bacteriophages Methods,” in Molecular Biology. Eds. Clokie M. R. J. Kropinski A. M. (Totowa, NJ: Humana Press), 3–14. doi: 10.1007/978-1-60327-164-6_1 19066805

[B115] Zhang Q. Xing S. Sun Q. Pei G. Cheng S. Liu Y.. (2017). Characterization and Complete Genome Sequence Analysis of a Novel Virulent Siphoviridae Phage Against Staphylococcus Aureus Isolated From Bovine Mastitis in Xinjiang, China. Virus Genes 53, 464–476. doi: 10.1007/s11262-017-1445-z 28299517

